# Biocompatible Pickering Emulsions from *Andrias davidianus* Byproducts for Promoting Burn Wound Healing

**DOI:** 10.34133/bmr.0233

**Published:** 2025-08-19

**Authors:** Jianlin Luo, Mingbo Wang, Lulu Qiu, Qingqing Huang, Xiaoting Liu, Linying Hao, Ting Ye, Wencong Shang, Kaizhuo Wang, Hui Wang, Yonglu Meng, Kai Xu, Can Li

**Affiliations:** ^1^ Engineering Research Center for Conservation and Efficient Utilization of Mountainous Biological Resources in Guizhou Province, Guiyang Innovation Center for Industrial Technology in Ecological Fisheries, College of Biological and Environmental Engineering, Guiyang University, Guiyang 550000, Guizhou, P.R. China.; ^2^ Department of Oncology, The First Affiliated Hospital of Henan Medical University, Weihui 453100, Henan, P.R. China.; ^3^ Tyran (Guangzhou) Biotechnology Co. Ltd., Guangzhou 511447, Guangdong, P.R. China.; ^4^ Guizhou Moutai Brewery (Group) Health Care Wine Industry Co. Ltd., Zunyi 563000, Guizhou, P.R. China.

## Abstract

Biological dressings have emerged as a promising approach for effective wound treatment. However, despite extensive research, the fabrication of biomass-based dressings with antioxidant and anti-inflammatory properties, as well as high biocompatibility, remains a challenge. In this study, the byproducts of *Andrias davidianus* as raw materials were used to prepare a biomass-based Pickering emulsion. A stable emulsion was formed by homogenizing *A. davidianus* collagen (AD-SC) with liver oil (AD-LO). The antioxidant peptides (AD-BP) were then incorporated into the mixture, and a Pickering emulsion loaded with antioxidant peptides was successfully prepared. The stability of AD-PE was confirmed through storage, centrifugation, and ζ-potential analyses, and the emulsion exhibited the controlled release of the peptides. In vitro experiments confirmed that AD-PE exhibited marked antioxidant activity and high biocompatibility, with no cytotoxicity, and the promotion of cell migration. In addition, in vivo evaluations demonstrated that AD-PE accelerated wound healing by leveraging the synergistic effects of its components to reduce inflammation and mitigate oxidative damage. This work offers a novel approach for the biomedical application of AD-PE and a new strategy for the utilization of *A. davidianus* processing byproducts.

## Introduction

Burn injuries severely compromise the structural integrity and functionality of skin, initiating a complex wound healing process that encompasses 3 key phases: inflammation, proliferation, and maturation [1,2]. In the early stages of wound healing, various cytokines, chemokines, and growth factors are rapidly released, triggering the inflammatory phase. However, burn injuries frequently result in prolonged inflammatory responses, characterized by a sustained release of pro-inflammatory immune cells and cytokines, extending the inflammatory phase and hindering overall wound repair [3]. One major consequence of this prolonged inflammation is the excessive production of reactive oxygen species (ROS), which cause oxidative stress and cellular damage. Oxidative damage refers to the biochemical harm induced by elevated levels of ROS, which react with critical biomolecules such as lipids, proteins, and nucleic acids, leading to lipid peroxidation, protein denaturation, and DNA strand breaks [4]. This oxidative damage can disrupt the formation of new blood vessels, impair normal metabolic functions, and further prolong inflammation, ultimately delaying wound healing [5,6]. Therefore, effective wound management strategies must focus on scavenging ROS and reducing inflammation to accelerate the healing process. Current approaches often incorporate antibiotics and antioxidants into wound dressings to counteract elevated ROS levels and inflammation. However, infection and antibiotic resistance remain critical challenges in the field of wound care [7].

Natural biomaterials with intrinsic anti-inflammatory and antioxidant properties have attracted increasing attention to mitigate oxidative damage and address antibiotic resistance. For example, marine oils have demonstrated therapeutic potential in reducing inflammation due to their fatty acid composition, with fish oil widely used for treating inflammatory conditions [8]. Collagen, a crucial extracellular matrix (ECM) component [9], has achieved widespread use in tissue engineering, biomedicine, cosmetics, and food industry due to its excellent biocompatibility. Collagen-based dressings have proven effective in reducing wound inflammation and promoting angiogenesis [10]. However, collagen sourced from terrestrial animals may cause issues due to immunogenicity and the risk of cross-species transmission of diseases, such as prion diseases [11]. Consequently, aquatic collagen has emerged as a safer alternative due to its abundance and low risk of cross-species disease transmission. Bioactive peptides from marine sources, including tuna [12] and mussels [13], have demonstrated potent antioxidant properties, especially low molecular weight peptides with strong antioxidant activity [14]. Despite these advantages, collagen derived from aquatic species often contains lower proline and hydroxyproline levels, leading to reduced structural and thermal stability compared with mammalian collagen and limiting its use in tissue engineering [14]. Chinese giant salamander (*Andrias davidianus*) is the largest and one of the oldest amphibians, with unique nutritional and medicinal benefits. Studies have shown that collagen extracted from *A. davidianus* has superior thermal stability, making it a viable alternative to mammalian and marine collagen for biomedical applications [15]. Due to their high viscosity and bioactivity, recent advancements have utilized the byproducts of *A. davidianus*, including skin secretions, to create innovative materials such as hemostatic powders [16], hemostatic sponges [17], bio-inks [18], biological adhesives [19], and hydrogels to promote bone regeneration [20]. However, other valuable byproducts, including *A. davidianus* skin and cartilage, have been underutilized, leading to resource waste. Notably, collagen extracted from *A. davidianus* skin has been identified as type I collagen, characterized by high proline content, with remarkable elasticity and thermal stability, making it a promising biomaterial for various applications [21].

Pickering emulsions offer an excellent option for developing advanced biomaterials that can effectively scavenge ROS and reduce inflammation [22]. Unlike traditional emulsions that rely on surfactants, Pickering emulsions are stabilized by solid nanoparticles, lowering their interfacial energy and resulting in enhanced stability [23]. This stabilization method also allows for improved responsiveness to external stimuli and enables the controlled release of bioactive substances, making these emulsions highly suitable for biomedical applications [24]. Studies have shown that microgel particles can effectively reduce oil–water interfacial tension [25], producing more stable Pickering emulsions with higher loading capacities than those stabilized by rigid particles [2]. These properties make Pickering emulsions ideal candidates for drug delivery and localized therapeutic administration [23].

**Fig. 1.**
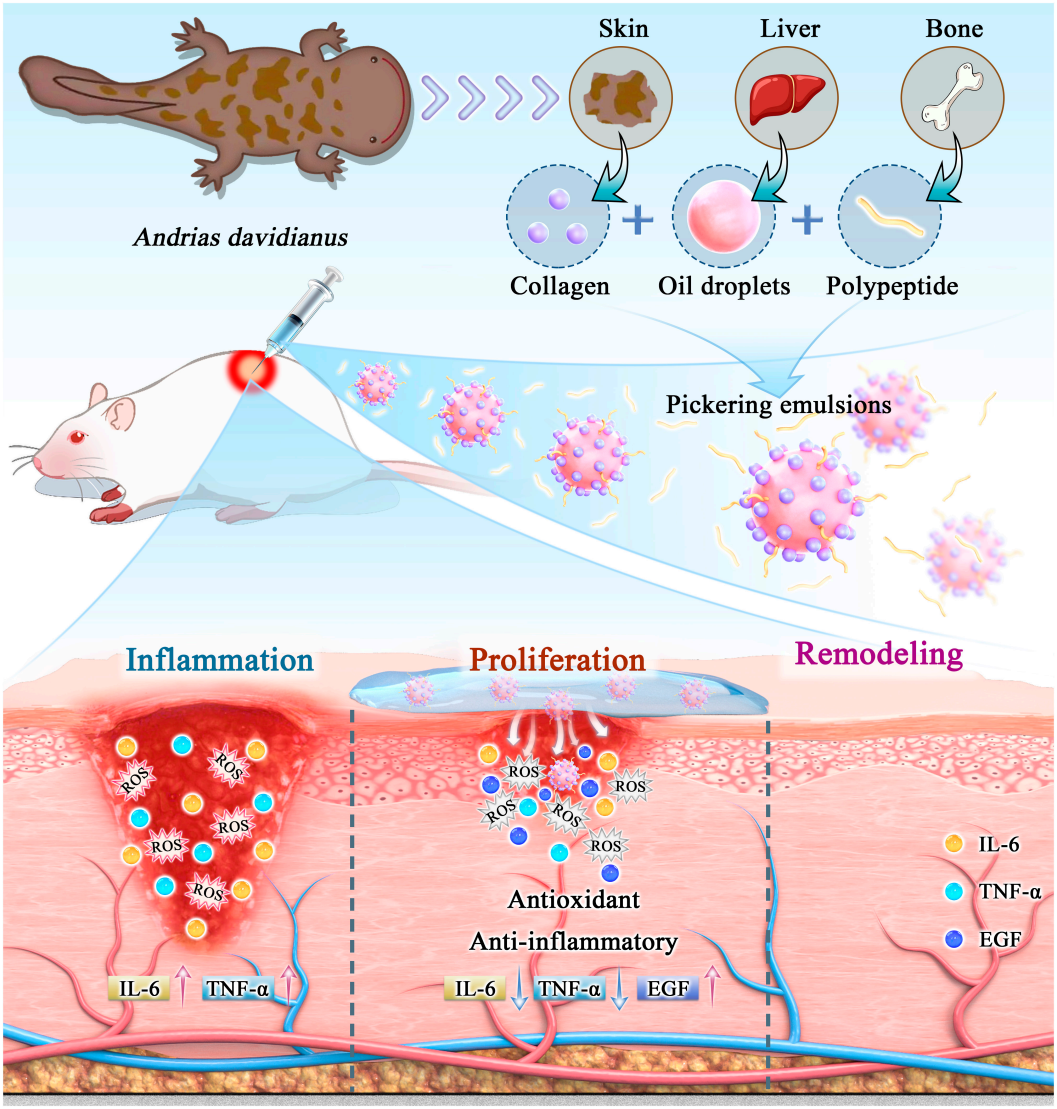
Preparation and application of Pickering emulsion. Preparation: Collagen was extracted from the skin, liver oil from the liver, and bone-derived peptides from the bones of the Chinese giant salamander (*A. davidianus*). A Pickering emulsion was subsequently prepared by homogenization. Application: The Pickering emulsion was applied to burn wounds, facilitating wound healing through its antioxidant and anti-inflammatory properties.

In this study, collagen was extracted from *A. davidianus* (AD-SC) skin, antioxidant peptides were isolated from its bones (AD-BP), and oil was obtained from its liver (AD-LO). AD-SC microgel solid particles were employed to stabilize AD-LO, leading to the preparation of a Pickering emulsion loaded with antioxidant peptides (Fig. 1). When applied to burn wounds, reduced inflammation and scavenged ROS were observed. This enhanced epidermal growth factor (EGF) secretion, promoted epidermal regeneration, and accelerated the wound healing process. This study presented a novel approach for the treatment of burns and scalds, offering a new strategy for the development of advanced biomaterials to meet the complex requirements of wound healing.

## Materials and Methods

### Materials

The Chinese giant salamander byproducts, including skin, bone, and liver, were obtained from healthy farm-raised *A. davidianus*, provided by Guizhou Qianmeng Agricultural Technology Co. Ltd. 2,2′-Azino-bis(3-ethylbenzothiazoline-6-sulfonic acid) (ABTS), 1,1-diphenyl-2-trinitrophenylhydrazine (DPPH), bovine serum albumin (BSA), physiological saline, phosphomolybdic acid solution, and Triton X-100 solution were purchased from Beijing Solarbio Science & Technology Co. Ltd. Nile Blue, Nile Red A, and Ponceau Red were obtained from Aladdin Reagent Co. Ltd. (Shanghai). The immunohistochemistry kits were sourced from Wuhan Boshide Biotechnology Co. Ltd. Jingwanhong ointment (JWH; a commercially available burn ointment) was provided by Guizhou Chenchun Pharmaceutical Chain Co. Ltd. All other reagents were supplied by Sinopharm Group Chemical Reagent Co. Ltd. All chemicals used in this study were of reagent grade.

### Extraction of collagen from Chinese giant salamander skin (AD-SC)

The collagen extraction method followed a previously described protocol [21]. First, the washed skins were immersed in a 10% isopropanol solution to remove fat. The defatted skins were then washed and treated with a 0.5 M sodium hydroxide solution to remove noncollagenous proteins. After further washing, the skins underwent enzymatic digestion with pepsin for 48 h. The resulting mixture was centrifuged at 4,000*g* for 15 min. Collagen was precipitated by adding NaCl to a final concentration of 0.9 M. The precipitated collagen was collected through centrifugation and further purified via dialysis with a 7-kDa molecular weight cutoff membrane for 48 h. Finally, the purified collagen was obtained after lyophilization (2.5 L FreeZone, Labconco, USA).

Scanning electron microscopy (SEM) analysis: For SEM analysis, the lyophilized collagen was sectioned and affixed to the sample stage using conductive adhesive. Subsequently, the samples were coated with a 10-nm layer of gold nanoparticles via ion sputtering. Finally, the surface and cross-sectional microstructures were observed at an accelerating voltage of 15 kV using a scanning electron microscope (Hitachi SU-8010, Japan).

### Extraction and characterization of antioxidative peptide from Chinese giant salamander bone (AD-BP)

The bones of the Chinese giant salamander were ground into powder and immersed in a 0.5 M sodium hydroxide solution with continuous stirring for 24 h to remove lipids. After thorough rinsing, the bone powder was transferred to a 0.5 M EDTA-2Na solution (pH 8.0) for decalcification over a period of 24 h. The resulting defatted and decalcified bone powder was suspended in water at a liquid-to-solid ratio of 5 mg/ml and enzymatically hydrolyzed using alkaline protease (Solarbio, Beijing, China) under magnetic stirring. The enzyme was inactivated by heating the solution to 100 °C for 10 min, followed by centrifugation at 10,000*g* for 10 min to collect the supernatant. The peptide fractions were separated using an ultrafiltration device (Shandong Bona Bio-Tech Group Co. Ltd., Jinan, China) and then lyophilized to obtain antioxidant peptides (AD-BP).

Fourier-transform infrared spectroscopy (FTIR) analysis: The AD-BP powder was mixed with potassium bromide (KBr) powder and compressed into thin slices under a pressure of 20 MPa for 3 min. FTIR analysis was conducted using an infrared spectrometer (Nicolet iS 10, Thermo Fisher Scientific, Waltham, MA, USA) in attenuated total reflectance (ATR) mode. Scans were performed at room temperature with a resolution of 4 cm^−1^, covering a wavenumber range of 400 to 4,000 cm^−1^. Each sample was scanned 32 times to ensure spectral accuracy.

ABTS radical scavenging activity assay: Samples and reference solutions (50 μl) with different mass concentrations (1, 2, 3, 4, and 5 mg/ml) were added to a 96-well plate along with 100 μl of ABTS working solution. The reaction was carried out in the dark at 25 °C for 30 min. Absorbance was measured at 734 nm using a microplate reader (MK3, Thermo Fisher Scientific, USA).

DPPH radical scavenging activity assay: The antiradical activity was evaluated using the DPPH assay. Samples and vitamin C solutions with varying concentrations (1, 2, 3, 4, and 5 mg/ml) were prepared. A 100-μl aliquot of each sample or control solution was added to a 96-well plate, followed by 100 μl of DPPH solution. The reaction was carried out in the dark at room temperature. After incubation, the absorbance was measured at 517 nm using a microplate reader (Thermo Fisher Scientific) to determine the scavenging activity.

Determination of hydroxyl radical scavenging ability: Samples of varying concentrations (1, 2, 3, 4, and 5 mg/ml) and control solutions (500 μl), ferrous sulfate solution (500 μl), and diluted hydrogen peroxide (1 ml) were mixed. The mixture was incubated in a water bath and centrifuged. The supernatant was transferred to a 96-well plate, and absorbance was measured at 510 nm using a microplate reader (Thermo Fisher Scientific).

### Extraction of oil from giant salamander liver (AD-LO)

The Chinese giant salamander liver was homogenized with phosphate-buffered saline (PBS) buffer. Neutral protease was added, and the mixture was enzymatically hydrolyzed at 50 °C and pH 10 for 3 h. The enzymatic reaction was terminated by heating the mixture at 100 °C for 10 min to deactivate the enzyme. Finally, the mixture was centrifuged at 10,000*g* for 10 min to obtain AD-LO.

Fatty acid analysis: Fatty acid analysis was conducted using a gas chromatograph (Agilent Technologies, Santa Clara, CA, USA) equipped with a capillary column (30 m × 0.25 mm × 0.25 mm). The injector and detector temperatures were set at 250 °C. The oven temperature was initially held at 50 °C for 1 min, then ramped up to 165 °C at 25 °C/min, followed by an increase to 190 °C at 1 °C/min, where it was maintained for 5 min. Finally, the temperature was raised to 230 °C at 5 °C/min and held until the analysis was completed.

### Preparation and characterization of Pickering emulsions

AD-SC was dissolved in 5 M acetic acid at a concentration of 50 mg/ml and dialyzed against ultrapure water for 3 d to remove residual acetic acid, forming an AD-SC gel. The gel was homogenized with ultrapure water to prepare solutions of varying concentrations (5, 10, 15, 20, and 25 mg/ml). For the emulsions, the AD-BP concentration was fixed at 5 mg/ml with an oil-to-water ratio of 2:8 (v/v). AD-LO and the prepared AD-SC solutions at different concentrations were combined to produce emulsions with varying AD-SC concentrations. Similarly, to explore the impact of oil-water ratios, emulsions were prepared with a fixed AD-SC concentration of 20 mg/ml and AD-BP at 5 mg/ml while varying the oil-to-water ratios (1:9, 2:8, 3:7, 4:6, and 5:5 v/v).

Stability of the Pickering emulsions: The storage stability of the samples was evaluated by storing them at room temperature for 30 d. The centrifugal stability was assessed by centrifuging the samples at 3,500*g* for 5 min.

Particle size analysis: The particle size distributions of AD-PE were determined using a laser particle analyzer with a detection range of 0.1 to 300 μm. Measurements were performed at 25 °C and repeated 3 times for each sample (BT-9300HT, Dandong Bettersize Instruments Ltd.).

ζ-Potential measurements: The emulsions were diluted 30-fold, and the ζ-potential was measured using a Zeta potential analyzer (Zetasizer Nano ZS90, Malvern Instruments Ltd., UK).

Rheological measurement: The apparent viscosity was measured using a rheometer (Malvern Instruments, Worcestershire, Malvern, UK) by scanning at a shear rate of 0.1 to 100 s^−1^. Each emulsion was tested 3 times at 25 °C, and the average values were reported.

Confocal laser scanning microscopy: Confocal laser scanning microscopy was utilized to observe the AD-SC particles and emulsions. For fluorescence staining, 20 μl of Nile Red and Nile Blue solutions (1 mg/ml each) were added to 1 ml of the emulsion and incubated overnight at 4 °C in the dark to ensure thorough staining. The stained sample was then carefully placed onto a concave confocal microscope slide for imaging. Using a 40× magnification objective lens, AD-SC particles were visualized under an excitation wavelength of 405 nm, while AD-LO was observed using an excitation wavelength of 633 nm. Images were captured using a laser scanning confocal microscope (Leica, Wetzlar, Germany).

### AD-BP sustained release

AD-BP was dissolved in distilled water and subsequently diluted to concentrations of 50, 25, 10, 5, and 0.25 mg/ml. The ultraviolet (UV) absorbance of these solutions was measured at 214 nm using a UV-visible spectrophotometer, with distilled water as the reference, to generate a standard curve. Following this, the Pickering emulsion was placed in a dialysis bag and immersed in PBS buffer solution (pH 7.4) at 37 °C with stirring. At designated time intervals, 5 ml of the sample solution was withdrawn to measure the release of AD-BP via UV spectrophotometry while simultaneously replacing the withdrawn volume with 5 ml of fresh PBS. A release curve of AD-BP in the Pickering emulsion was then plotted.

### In vitro biocompatibility

Preparation of extract :AD-PE and its individual components were immersed at a concentration of 10 mg/ml in RPMI 1640 medium supplemented with 10% fetal bovine serum (FBS) and 1% penicillin/streptomycin at 4 °C for 24 h. After the incubation period, the samples were filtered out to remove any remaining solid materials, and the resulting solution was collected as the extract.

Cell viability: The cytotoxicity of the PE and its components was evaluated in vitro using the Cell Counting Kit-8 (CCK-8) assay. L929 fibroblasts were seeded into a 96-well plate at a density of 5 × 10^3^ cells per well in 100 μl of culture medium and incubated for 24 h. Subsequently, the culture medium was replaced with 100 μl of the extraction medium from the samples, and the cells were incubated for additional 24 h. Afterward, the cells were washed with PBS and incubated with 100 μl of RPMI 1640 containing 10% CCK-8 reagent (Boxbio, Beijing, China) per well for 1 h. Finally, the absorbance at 450 nm was measured using a microplate reader (MK3, Thermo Fisher Scientific, USA).

Cell migration assay: L929 cells were seeded into 6-well plates at a density of 5 × 10^4^ cells per well and incubated at 37 °C in a 5% CO₂ incubator for 24 h. A sterile pipette tip was used to create a scratch perpendicular to a sterile ruler, generating a consistent wound area. The cells were then rinsed twice with sterile PBS to remove debris. Subsequently, 2 ml of serum-free RPMI 1640 medium containing the sample extract was added to each well, while the control group received serum-free RPMI 1640 medium without the extract. After 24 h of incubation, images of the scratched areas were captured. The wound widths were measured using ImageJ software, and the wound healing percentage was calculated using the following formula:

$$\begin{array}{c} \text{Wound healing percentage}=\\ \left[1-\frac{\text{Width of wound}\;at\;24\;h}{\text{Width of wound}\;at\;\text{time}\;=0}\right]\times 100\% \end{array}$$ (1)

Intracellular ROS scavenging activity: L929 cells were seeded into a 96-well plate at a density of 1 × 10^4^ cells per well in 100 μl of complete RPMI 1640 medium and incubated overnight at 37 °C with 5% CO₂. The cells were then treated with extraction solutions of AD-BP, AD-LO, AD-SC, or AD-PE for 24 h. After incubation, 100 μl of 200 μM H₂O₂ was added to each well to induce oxidative stress, followed by 4-h incubation. Next, 10 mM of the ROS-sensitive fluorescent probe 2′,7′-dichlorodihydrofluorescein diacetate (DCFH-DA) was added to each well, and the plate was incubated for an additional 30 min. The cells were washed thoroughly with PBS to remove excess probe. Intracellular ROS levels were then quantified using a multifunctional microplate reader, measuring fluorescence intensity.

Intracellular malondialdehyde (MDA) level: MDA levels were determined using a commercial MDA Content Assay Kit (BC0025, Solarbio, Beijing, China) according to the manufacturer’s instructions. Briefly, L929 cells were seeded in 6-well culture plates at a density of 1 × 10^6^ cells per well and incubated overnight. The cells were then treated with the extraction solutions for 24 h and subjected to 200 μM H₂O₂ stimulation for additional 4 h. Following treatment, the cells were harvested and lysed using the extraction buffer. MDA content was measured by reacting with thiobarbituric acid, which forms a red-colored complex. The absorbance of the resulting complex was quantified at 532 nm using a microplate reader (MK3, Thermo Fisher Scientific, USA).

Superoxide dismutase (SOD) activity: SOD activity was measured using the SOD assay kit (BC5165, Solarbio, Beijing, China) according to the manufacturer’s instructions. L929 cells were treated with the corresponding samples, and cellular SOD activity was assessed by measuring the inhibition of WST-1 reduction, which reflects the scavenging of superoxide radicals. The absorbance of the reaction mixture was measured at 450 nm, and the results were expressed as units of SOD activity per milligram of protein.

Enzyme-linked immunosorbent assay (ELISA) procedure for interleukin-6 (IL-6) and tumor necrosis factor-α (TNF-α): Raw264.7 cells were seeded in 6-well culture plates at a density of 1 × 10^6^ cells per well and incubated overnight in RPMI 1640 medium supplemented with 10% FBS. After the initial incubation, the cells were treated with the corresponding extracts for 24 h, followed by stimulation with 100 ng/ml lipopolysaccharide (LPS) for an additional 24 h. After treatment, the culture supernatants were collected. The levels of IL-6 and TNF-α in the supernatants were determined using commercially available ELISA kits (SEKM-0007 and SEKM-0034, Solarbio, Beijing, China).

### In vivo deep second-degree burn wound healing

Sprague–Dawley rats (male, 7 to 8 weeks of age, 200 to 250 g) were anesthetized via intraperitoneal injection of 10% chloral hydrate (300 mg/kg) [26]. After disinfecting the back with alcohol, a circular wound with a diameter of 1.2 cm was created. The negative control (normal saline), positive control (JWH ointment), and sample groups (AD-PE and its components) were applied once daily for 19 consecutive days. All animal procedures were conducted in accordance with the guidelines approved by the Guiyang University Experimental Animal Ethics.

The burn wound areas of the rats were recorded on days 0, 7, 14, and 19. The wound area was then measured by ImageJ to calculate the wound healing rate (WHR) following: WHR (%) = (*A_i_* − *A_f_*)/*A_i_* × 100, where *A_i_* and *A_f_* represent the burn wound area at day 0 and day *n*, respectively.

Histopathological analysis: The skin surrounding the wounds was excised into 1 × 1 cm^2^ pieces, fixed in 4% paraformaldehyde, and embedded in paraffin. The embedded tissues were sectioned into 5-μm-thick slices and stained with hematoxylin and eosin (H&E; Solarbio, Beijing, China) or Masson’s trichrome staining kit (Solarbio, Beijing, China) for histopathological analysis. Histological images were captured using an Olympus BX43 upright microscope (Tokyo, Japan).

Determination of EGF concentration: The wound tissues were carefully rinsed with ice-cold saline and subsequently immersed in PBS at a 1:10 ratio. The samples were homogenized on ice and centrifuged at 5,000*g* for 10 min. The EGF concentration in the supernatant was then measured using an ELISA kit (R&D Systems, Minneapolis, MN, USA).

Immunohistochemistry: For immunohistochemical staining of CD31, endogenous peroxidase activity was blocked by incubating the tissue sections in 3% hydrogen peroxide at room temperature for 30 min, following heat-induced antigen retrieval. The slides were then blocked with 10% normal goat serum for 1 h, followed by incubation with anti-CD31 antibody (Proteintech, Wuhan, China) at 4 °C overnight. Subsequently, the sections were incubated with an horseradish peroxidase-conjugated secondary antibody (Proteintech, Wuhan, China) for 1 h at room temperature, visualized using 3,3′-diaminobenzidine (DAB; Beyotime Biotechnology, Guangzhou, China), and counterstained with hematoxylin. Images were captured using an Olympus BX43 upright microscope (Tokyo, Japan).

Immunofluorescence staining: On the fifth day, wound tissue samples were collected for immunofluorescence staining to analyze the expression of IL-6 and TNF-α. The tissues were fixed in 4% paraformaldehyde, embedded in optimal cutting temperature (OCT) compound, and sectioned into 8-μm slices using a cryostat microtome (Leica, Wetzlar, Germany). The sections were permeabilized with 0.3% Triton X-100, blocked with 10% normal goat serum, and incubated overnight at 4 °C with primary antibodies against IL-6 (Proteintech, Wuhan, China) and TNF-α (Proteintech, Wuhan, China). After washing, the sections were incubated with Alexa Fluor 488-conjugated secondary antibody (Cell Signaling Technology, Shanghai, China) in the dark for 1 h at room temperature. Nuclei were counterstained with 4′,6-diamidino-2-phenylindole. Finally, the stained sections were imaged using a fluorescence microscope (Carl Zeiss, Oberkochen, Germany). The fluorescence intensity was quantitatively analyzed using ImageJ (NIH, Bethesda, MD, USA) to assess protein expression levels.

ROS: On the 5th day post-burn, skin samples from the wound site of rats were collected to assess the ROS content. The fresh tissue samples were washed with PBS, and then 50 mg of tissue was weighed and placed in 1 ml of homogenization buffer A. The tissue was thoroughly homogenized using a homogenizer. In a 96-well plate, 20 μl of the homogenate and 2 μl of dihydroethidium (DHE; Yeasen, Shanghai, China) solution were added, and the mixture was thoroughly mixed by pipetting. The plate was incubated at 37 °C in the dark for 30 min. Fluorescence intensity was measured using a fluorescence microplate reader with an excitation wavelength of 488 nm and an emission wavelength of 610 nm.

### Statistical analysis

All data are presented as mean ± SD from at least 3 independent experiments. Statistical significance was assessed using 1-way or 2-way analysis of variance (ANOVA) followed by Tukey’s post hoc test, as appropriate. Differences were considered statistically significant at *P* < 0.05.

## Results and Discussion

### Preparation and characterization of AD-SC and AD-BP

**Fig. 2.**
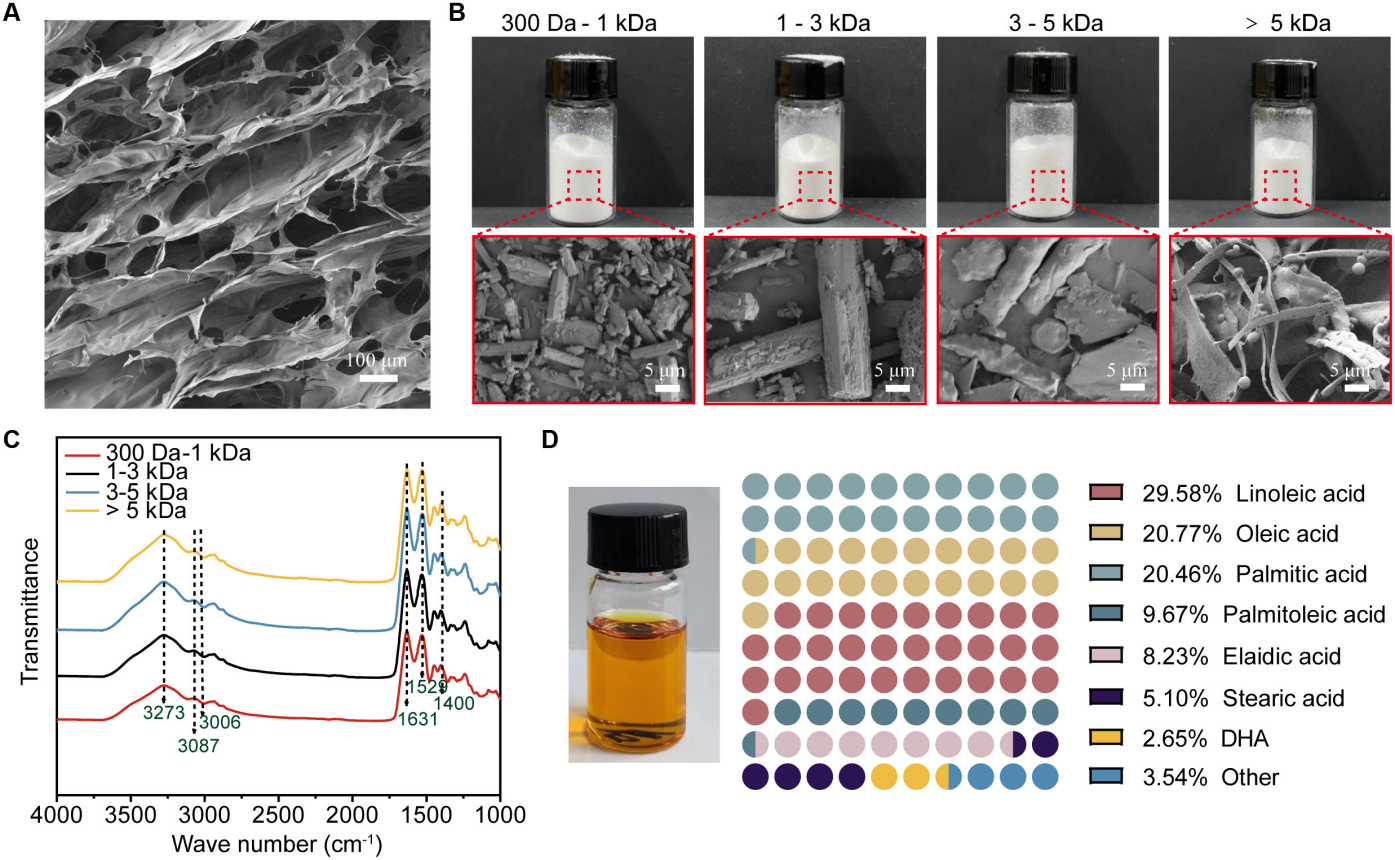
Image and structural characterizations of AD-SC, AD-BP, and AD-LO (*n* = 3). (A) SEM image of AD-SC.(B) SEM image of AD-BP with different molecular size. (C) FTIR spectra of the AD-BP with different molecular size. (D) Photograph and fatty acid species distribution of AD-LO.

The porous matrix structures of biomaterials play a crucial role in supporting cell proliferation and nutrient distribution and enhancing cell adhesion, facilitating vascularization and epithelial regeneration [27]. In this study, SEM analysis revealed that collagen extracted from AD-SC exhibited a porous, fibrous 3-dimensional structure (Fig. 2A). This structure provided a high surface area that promoted cell adhesion, migration, and growth while also serving as a potential drug delivery vehicle to enhance the biological functionality of the material [28]. In addition, the peptides possessed rich functional side chains, a diverse range of hydrophilic/hydrophobic characteristics, and the ability to form stable secondary structures. These properties allowed them to self-assemble into various ordered nanostructures [29]. The antioxidant peptides, derived from giant salamander bone, appeared as a powdery material (Fig. 2B), where the layered structure diminished as the molecular weight decreased. As the degree of hydrolysis increased, AD-BP was hydrolyzed into rod-like and granular structures, making it more suitable for drug delivery [30]. In the FTIR spectrum of AD-BP (Fig. 2C), the broad absorption band between 3,750 and 3,000 cm^−1^ is attributed to O–H and N–H stretching vibrations, indicating the presence of hydroxyl and amine groups as well as extensive intra- and intermolecular hydrogen bonding among peptide chains. The characteristic peak observed at approximately 1,631 cm^−1^ corresponds to the amide I band, associated with the C=O stretching of peptide bonds, while the peak at 1,529 cm^−1^ reflects the amide II band, mainly due to N–H bending and C–N stretching, confirming the polypeptide structure. Additionally, the band near 1,400 cm^−1^ can be ascribed to symmetric stretching of carboxylate groups (–COO^−^) from acidic amino acid residues. These –COO^−^ groups are potential chelating sites for ferrous ions (Fe^2+^), which can inhibit metal-catalyzed oxidative reactions, thereby enhancing the antioxidant capacity of the peptides. Notably, changes in peak intensity among different molecular weight fractions reflect variations in functional group content, secondary structure, and possible aggregation states, all of which may influence their biological activity [31]. AD-LO (Fig. 2D) exhibited a bright color and was free of noticeable impurities, containing a total of 70.81% oleic acid, linoleic acid, and palmitic acid. Previous studies indicated that palmitic acid can exhibit anti-inflammatory, antioxidant, and immune-enhancing properties [32], while linoleic acid can enhance immune function, promote cell division, and reduce inflammatory responses [33]. Consequently, AD-LO offers substantial potential in reducing inflammation and oxidative stress during the wound healing process, making it a critical component for promoting tissue regeneration.

### Radical scavenging activity of AD-BP

**Fig. 3.**
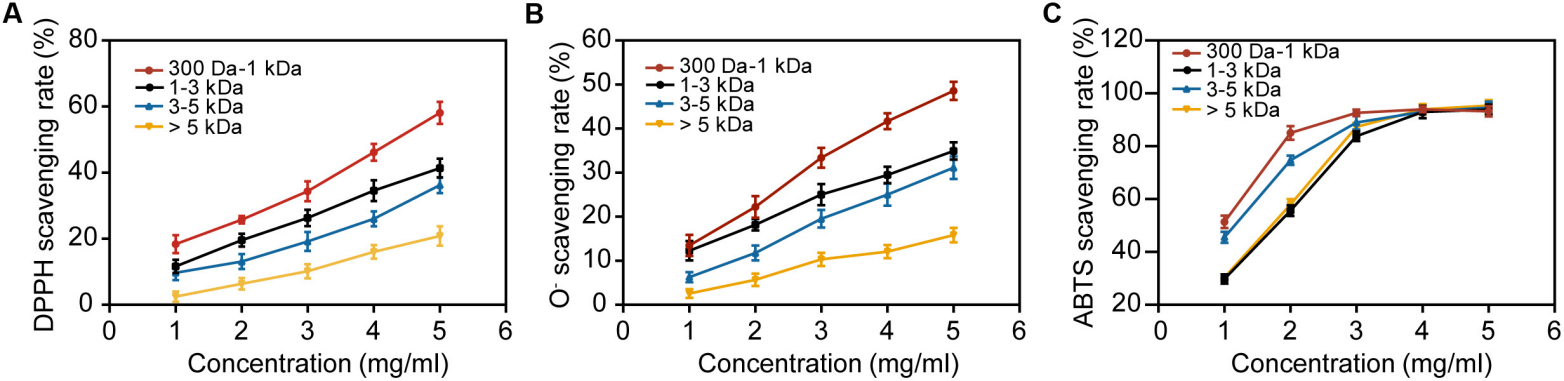
Scavenging capability of AD-BP with different molecular size to oxidants of (A) DPPH, (B) O_2_^−^, and (C) ABTS.

The hydrolysate of giant salamander bones was separated via ultrafiltration into AD-BP fractions of varying molecular weights, which all demonstrated the ability to reduce superoxide anion radical production and scavenge DPPH and ABTS radicals. A clear dose-dependent relationship was observed (Fig. 3A to C), with lower molecular weight peptides exhibiting greater antioxidant activity compared with higher molecular weight peptides [34]. At a concentration of 5 mg/ml, AD-BP with a molecular weight of 300 Da to 1 kDa achieved a 47.36% scavenging rate for superoxide anions (O₂^−^), a 59.06% scavenging rate for DPPH radicals, and greater than 90% scavenging rate for ABTS radicals. These findings indicated that AD-BP possessed potent antioxidant activity, especially in neutralizing ROS at high concentrations, which was critical for mitigating oxidative stress in wound environments and promoting wound healing. This antioxidant capacity made AD-BP an effective component for enhancing wound care formulations aimed at reducing oxidative damage and accelerating tissue regeneration.

### Preparation and stability analysis of AD-PE

**Fig. 4.**
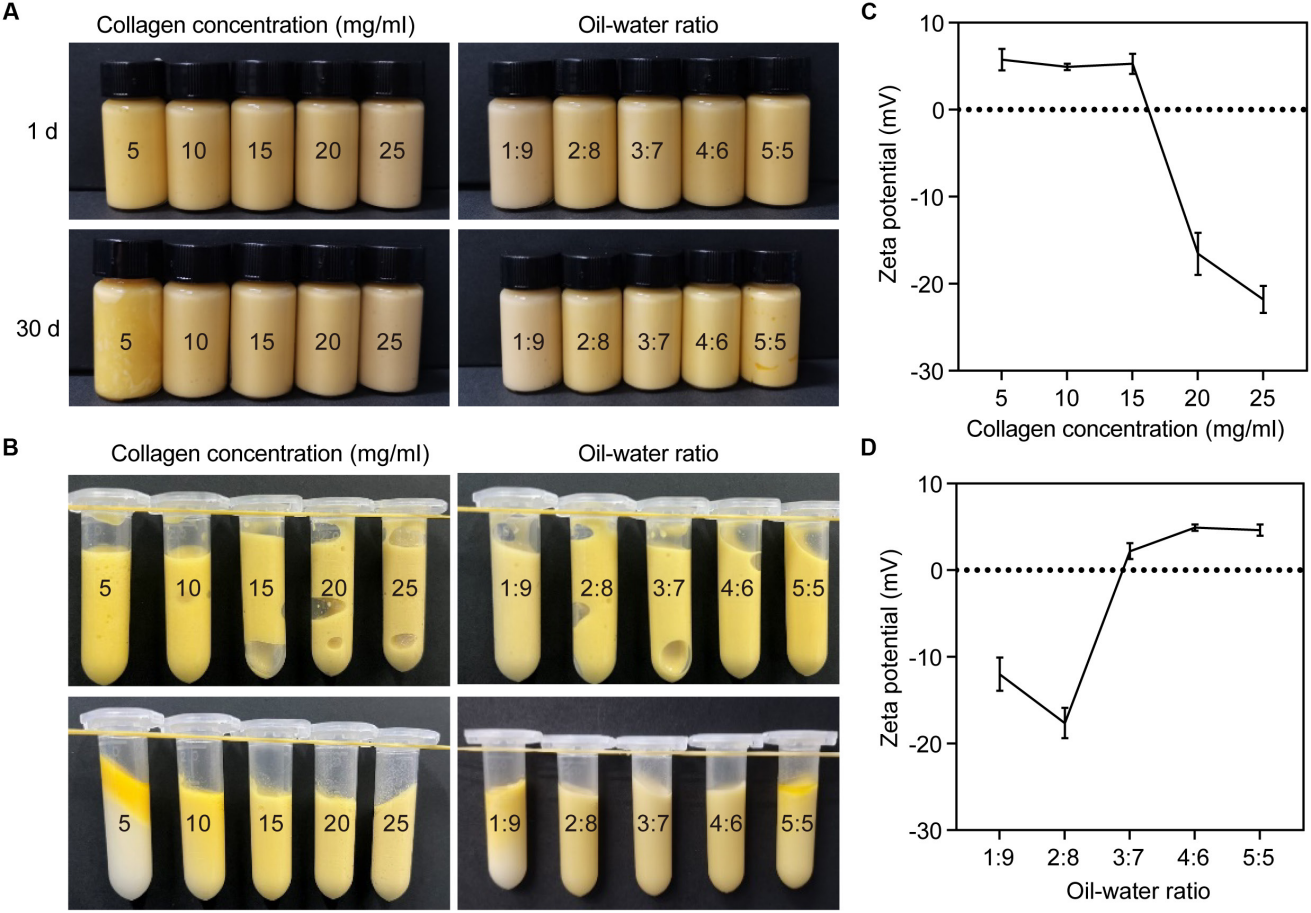
Effect of AD-SC concentration and different oil/water ratios on the storage stability of (A) AD-PE after 30 d and (B) centrifugation, and their impact on (C and D) AD-PE ζ-potential.

The stability of the AD-PE in terms of storage, centrifugation, and ζ-potential was found to be closely associated with the concentration of solid particles (AD-SC) and oil/water ratio [35]. At an AD-SC concentration of 5 mg/ml, distinct oil separation was observed in AD-PE after 30 d of storage (Fig. 4A) and centrifugation (Fig. 4B). A similar trend was also observed in emulsions with a high oil/water ratio, likely due to an insufficient concentration of AD-SC, which led to pronounced hydrophobic interactions between the oil droplets. This resulted in droplet aggregation, flocculation, and subsequent phase separation [36]. As the AD-SC concentration increased, no phase separation was observed following centrifugation. At different oil/water ratios, substantial phase separation occurred at a ratio of 1:9 post-centrifugation, which was likely due to the small oil phase leading to AD-SC aggregation. Conversely, when the oil phase was too high (5:5), oil separation occurred under entrifugation. These findings suggested that both insufficient solid particle concentration and excessive oil phase negatively impacted AD-PE stability. Thus, AD-PE prepared with an AD-SC concentration of greater than 10 mg/ml and oil/water ratio between 2:8 and 4:6 demonstrated superior stability.

The ζ-potential serves as a key indicator of emulsion stability, as its magnitude can influence the ability of the dispersion system to maintain stability over time [37]. As the concentration of AD-SC increased, the absolute value of the ζ-potential for AD-PE also increased, reaching approximately −23 mV at a concentration of 25 mg/ml (Fig. 4C). This increase in AD-SC concentration mitigated repulsive electrostatic interactions between the droplets, thereby enhancing the stability of AD-PE. By contrast, at oil/water ratios of 1:9 and 2:8 (Fig. 4D), the ζ-potential values were −11.34 and −18.3 mV, respectively, indicating optimal stability at these ratios. However, a higher oil phase ratio diminished the emulsion stability, as evidenced by the reduced ζ-potential values. These results suggested that appropriate AD-SC concentrations and oil/water ratios were essential for achieving stable emulsions.

### Particle size of AD-PE

**Fig. 5.**
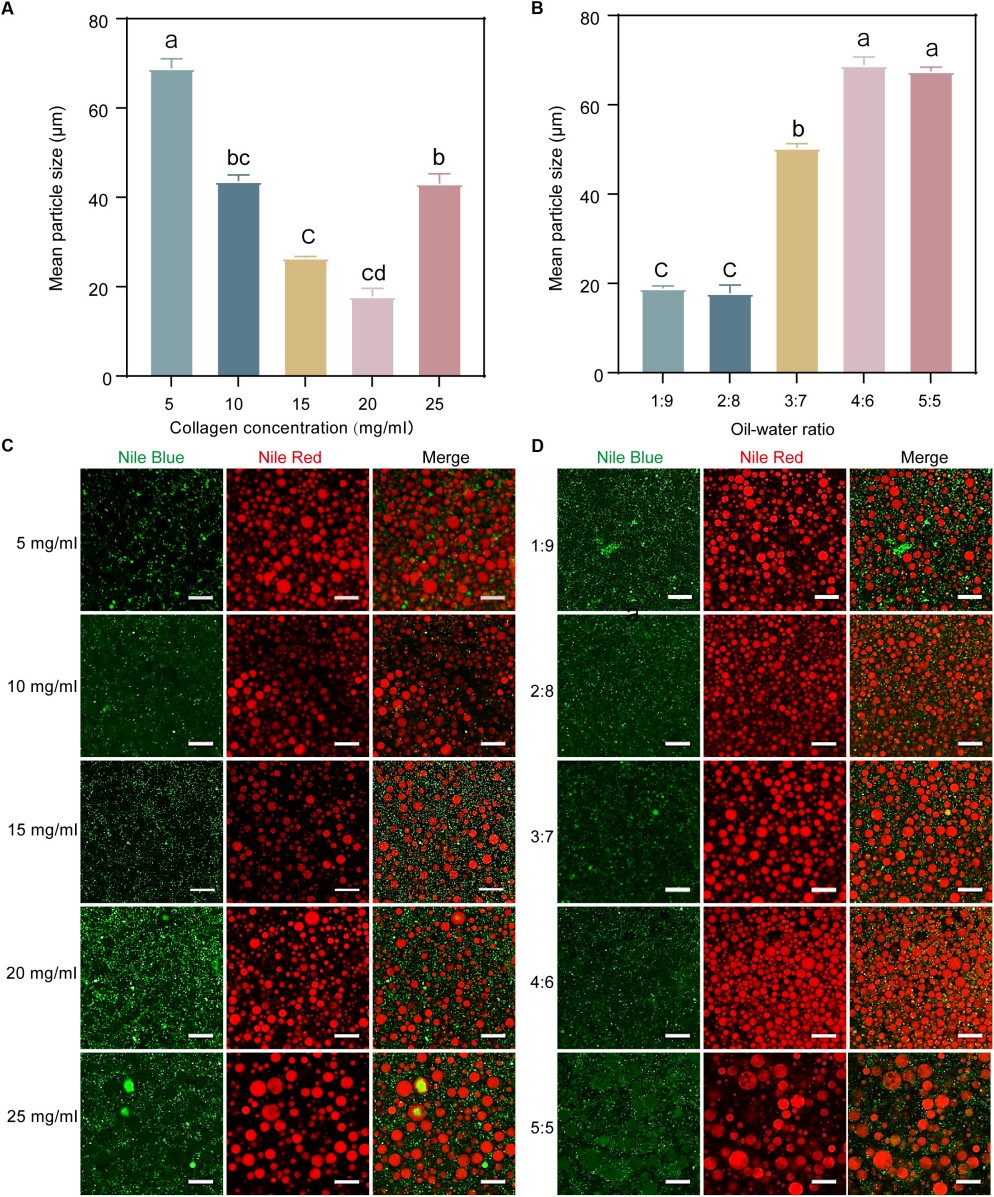
Effect of (A) AD-SC concentration and (B) different oil/water ratios on the particle size of AD-PE, measured using a laser diffraction particle size analyzer. (C and D) Laser confocal microscopy images of AD-PE prepared with different AD-SC (C) and oil-water ratios (D). AD-SC was stained with Nile Blue (green), and AD-LO was stained with Nile Red (red). Scale bar, 50 μm. Different letters indicate the significant difference (*P* < 0.05).

The particle size of an emulsion plays a crucial role in its stability, with smaller particles contributing to improved stability [38]. To evaluate the impact of AD-SC concentration and the oil/water ratio on the particle size of AD-PE, the oil/water ratio was fixed at 2:8 (Fig. 5A and B). Using a laser diffraction particle size analyzer, it was found that at an AD-SC concentration of 20 mg/ml, the droplet size reached its minimum, averaging 17.88 μm. This reduction in size was attributed to the higher AD-SC concentration, which increased steric hindrance between the droplets, thus limiting droplet coalescence. As a result, smaller droplets formed, contributing to a more stable emulsion [39].

Laser confocal microscopy provided further insights into the emulsion’s type and microstructure. The results showed that the red AD-LO droplets (red) were covered by AD-SC (green), confirming the formation of an oil-in-water emulsion (Fig. 5C and D). Furthermore, as the concentration of AD-SC increased, larger droplets gradually decreased, leading to a reduction in the average particle size (Fig. 5C). This finding corroborates the results from the particle size experiments. Additionally, as the oil/water ratio increased, the emulsion became more compact. At the ratio of 4:6, coalescence of droplets was observed (Fig. 5D), which may be due to insufficient AD-SC to adsorb onto the increased oil phase, leading to droplet aggregation [40]. Eventually, at an oil/water ratio of 5:5, larger droplets formed, resulting in an increase in the emulsion’s particle size (Fig. 5D).

### Rheological properties and sustained release performance of AD-PE

**Fig. 6.**
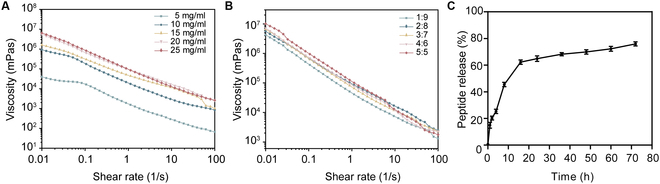
Effect of (A) AD-SC concentrations and (B) different oil/water ratios on the rheological properties of the AD-PE. (C) AD-BP sustained release curve in AD-PE.

According to Stokes’ law, increased emulsion viscosity will slow down droplet settling or flotation, thereby improving overall emulsion stability [38]. In the shear rate range of 10 to 100 s^−1^, all AD-PE formulations exhibited decreasing viscosity with increasing shear rate, demonstrating characteristic shear-thinning behavior (Fig. 6A and B). The 3-dimensional network structure of AD-SC contributed to the formation of a semi-solid emulsion gel with enhanced viscosity. The viscosity increased as the AD-SC concentration increased, forming a gel-like structure that increased resistance to droplet movement, thereby enhancing emulsion stability [35]. The viscosity of the emulsion played a crucial role in wound healing by enhancing its adhesion to the wound surface. Higher viscosity allowed for better contact with the wound, forming a stable protective barrier that not only secured the emulsion in place but also prolonged the retention of bioactive components. This extended exposure enhanced localized therapeutic efficacy, promoting more effective wound healing. Taking these factors into account, an AD-SC concentration of 20 mg/ml and oil-to-water ratio of 2:8 were selected as the optimal formulation for preparing AD-PE, making this formulation highly suitable for burn healing applications.

According to previous research, the AD-PE formulation with an AD-SC concentration of 20 mg/ml and oil/water ratio of 2:8 demonstrated excellent stability, while the microgel structure of AD-SC facilitated the controlled release of bioactive peptides [41,42]. The release process followed a 2-phase model, namely, an initial burst release followed by a sustained release (Fig. 6C). During the first 16 h, rapid burst release occurred, with a total release of 62.28%, primarily attributed to the AD-BP located on or near the droplet surfaces. After the initial phase, the sustained release began, gradually increasing to 75.94% by 72 h, with three-quarters of total release occurring between 16 and 72 h. These findings indicated that the AD-PE system could achieve controlled peptide release, aiding in the scavenging of ROS from wounds, and thereby enhancing its potential application in burn treatment.

### Biocompatibility, cell migration, ROS scavenging, and anti-inflammatory properties of AD-PE

**Fig. 7.**
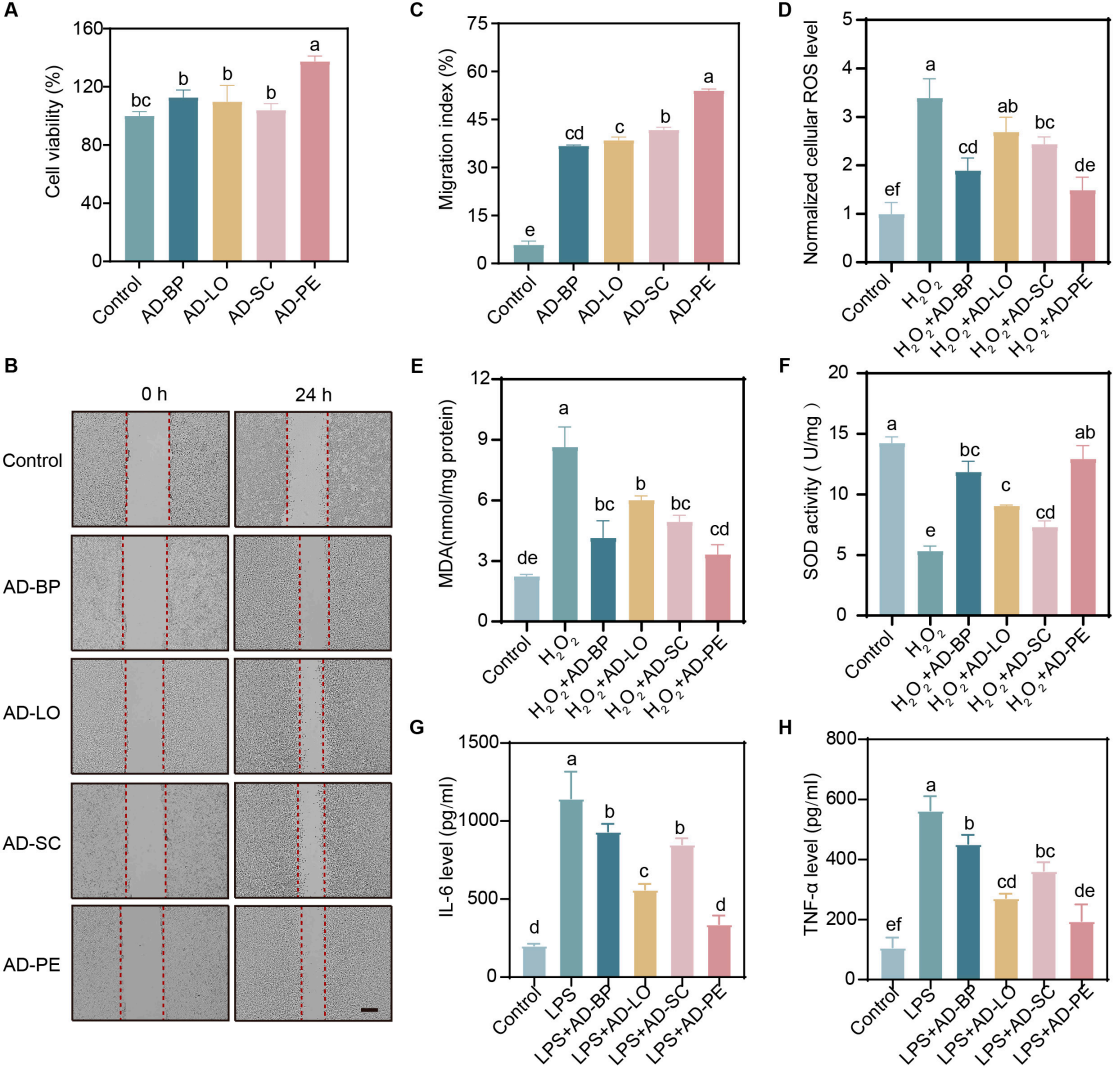
(A) L929 cells were incubated with extracts from various AD-derived components for 24 h, and cell viability was assessed using the CCK-8 assay. (B) L929 cells were treated with different extracts for 24 h, and cell migration was evaluated by scratch assay. Scale bar, 200 μm. (C) Cell migration index calculated from (B). L929 cells were exposed to different extracts and subjected to H_2_O_2_ stimulation. Cellular levels of ROS (D), MDA (E), and SOD activity (F) were measured. Raw264.7 cells were treated with different extracts followed by LPS stimulation, and IL-6 (G) and TNF-α (H) levels in the culture supernatant were quantified using ELISA. Different letters indicate the significant difference (*P* < 0.05).

The biocompatibility of AD-PE and its individual components was assessed using the CCK-8 assay. The results revealed that after 24 h of treatment, neither AD-PE nor its components exhibited cytotoxic effects on L929 cells. Furthermore, the treatments promoted varying degrees of cell proliferation, with AD-PE showing the most pronounced enhancement (Fig. 7A), suggesting that AD-PE not only is cytocompatible but also supports cellular growth, making it suitable for wound healing applications. Cell migration is a critical parameter for tissue regeneration and wound healing [43]. The effects of AD-PE and its components on cell migration were determined by scratch assay. The results showed that the cell migration rate of L929 cells treated with AD-PE and its components was significantly higher compared to the control group, with AD-PE showing the most pronounced enhancement (Fig. 7B and C). This further confirmed the excellent cytocompatibility of AD-PE.

Importantly, the observed biocompatibility of AD-PE may be closely associated with its antioxidant activity. Excessive intracellular ROS levels are known to impair cell viability and migration, leading to poor wound healing outcomes. By mitigating oxidative stress, antioxidant materials can help maintain a favorable cellular microenvironment, thus supporting normal cellular functions [44]. ROS are known to contribute to inflammation and delay wound healing. However, high concentrations of ROS generated under oxidative stress could exacerbate an inflammatory response and delay the wound healing process [45]. To evaluate the ROS scavenging ability of AD-PE and its components, L929 cells were exposed to H_2_O_2_-induced oxidative stress. The results revealed that AD-PE and its components effectively reduced intracellular ROS levels, with AD-PE showing the strongest effect (Fig. 7D). Additionally, the results of MDA and SOD activities (Fig. 7E and F) were consistent with the ROS scavenging findings. MDA levels, which are indicative of lipid peroxidation and oxidative stress, were significantly reduced in cells treated with AD-PE and its components, particularly AD-BP (Fig. 7E). In parallel, SOD activity, a key antioxidant defense mechanism, was significantly enhanced in the AD-PE-treated cells (Fig. 7F). Together, these results indicate that AD-PE and its constituents mitigate oxidative stress by lowering intracellular ROS and MDA levels while enhancing SOD activity. This antioxidant action is closely associated with improved cell viability and migration, suggesting that the ROS scavenging ability of AD-PE is a key factor contributing to its cytocompatibility and regenerative potential.

Furthermore, the inflammatory responses were evaluated by measuring the levels of IL-6 and TNF-α in the culture supernatant after LPS stimulation. Both IL-6 (Fig. 7G) and TNF-α (Fig. 7H) levels were significantly reduced in Raw264.7 cells treated with AD-PE and its components compared to the control group, indicating that AD-PE has potent anti-inflammatory effects. This suggests that AD-PE not only alleviates oxidative stress but also modulates inflammatory responses, further supporting its potential in promoting wound healing.

### In vivo wound healing performance of AD-PE

**Fig. 8.**
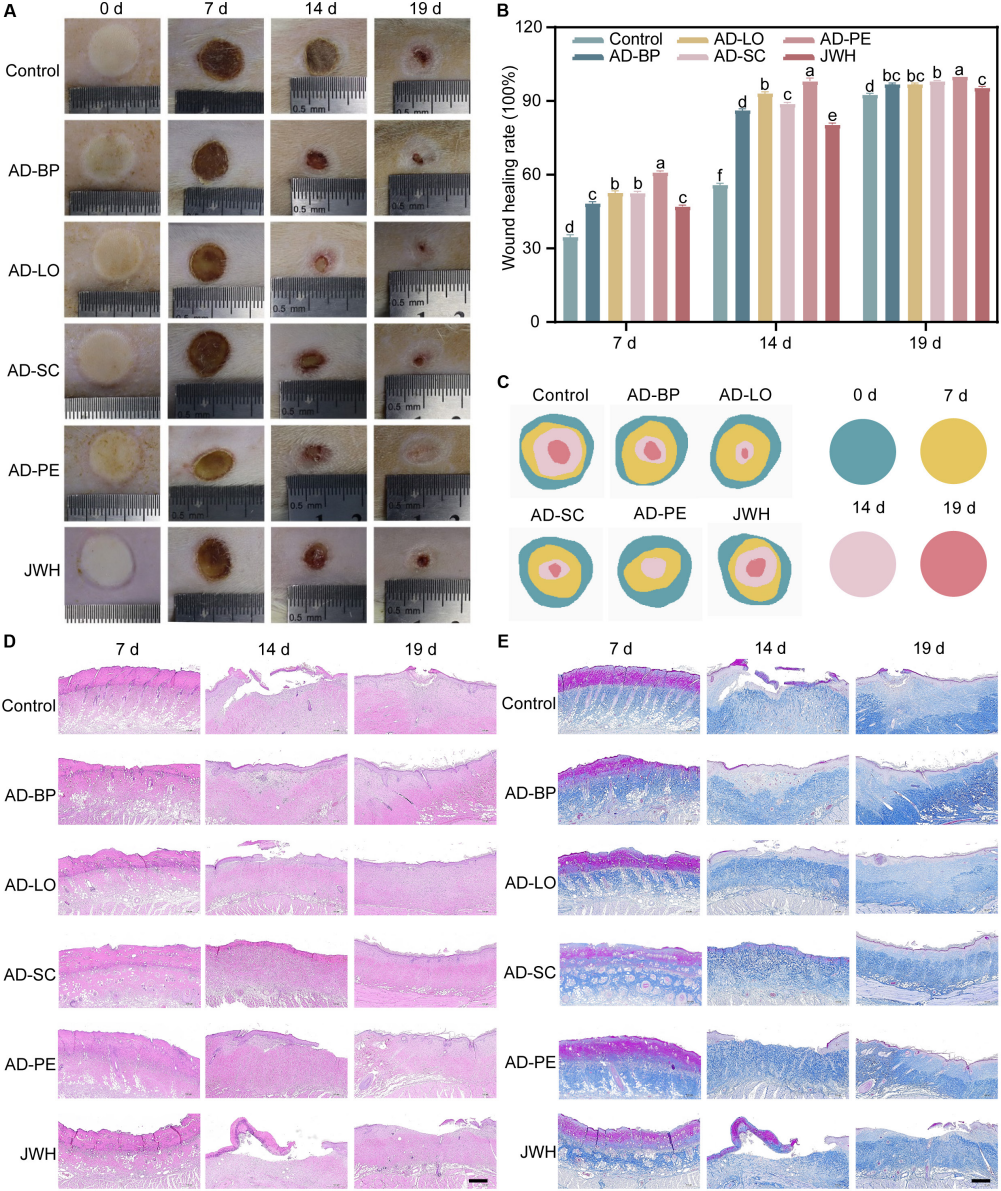
(A) Representative digital images of wounds, (B) quantitative analysis of wound closure rates based on wound area, (C) relative wound outlines, and (D) H&E (E) and Masson staining images of skin wound tissues following different treatments on days 7, 14, and 19. Scale bar, 500 μm. Different letters indicate the significant difference (*P* < 0.05).

A deep second-degree burn wound model with a diameter of 1.2 cm was established in rats to evaluate the in vivo wound healing effects of AD-PE and its components (Fig. 8A to C). After 7 d of treatment, the edges of the scabs in the AD-PE and JWH groups started to lift and detach, with the AD-PE group showing a more pronounced scab lifting and a higher rate of wound contraction compared with the other groups. This enhanced wound contraction was attributed to the excellent anti-inflammatory and antioxidant properties of the components, with AD-PE exhibiting a synergistic effect in promoting wound healing. After 14 d of treatment, noticeable differences in wound appearance were observed, where the healing rate in the AD-PE group reached 96.71%, compared with 80.4% in the JWH group, while the control group had just entered the contraction healing phase with a healing rate of only 55.95%. By day 19, the wounds in the AD-PE group had fully healed, while the healing rates for the JWH and control groups were 95.49% and 92.64%, respectively. These results demonstrated that AD-PE promoted wound healing and shortened the healing time.

In burn injuries, inflammation plays a key role in the underlying pathophysiology. Inflammatory signals stimulate fibroblast proliferation, which is essential for the production of ECM [46]. However, excessive inflammation can damage healthy tissue and delay the healing process [3]. Histopathological analysis of wounds at different time points was conducted using H&E and Masson staining (Fig. 8D). On day 7 post-burn, the AD-PE group only contained a few inflammatory cells, while the control and JWH groups displayed a significant number of inflammatory cells. By day 14, the control and JWH groups had thicker scabs with remaining inflammatory cells, while the AD-PE group showed visible epidermal growth. On day 19, the control and JWH groups contained thickened epidermal tissue, while the AD-PE group had nearly completed the healing process, with evidence of vascular and follicular regeneration, indicating good wound recovery. Masson staining (Fig. 8E) further demonstrated that by day 7 post-burn, the AD-PE group had considerable more collagen fiber deposition compared with the control group. By day 19, collagen in both the dermal and epidermal layers of the AD-PE group was well organized, with newly formed blood vessels and hair follicles, suggesting that AD-PE exhibited superior burn healing properties compared with the other groups.

### The molecular mechanism of wound healing induced by AD-PE in vivo

**Fig. 9.**
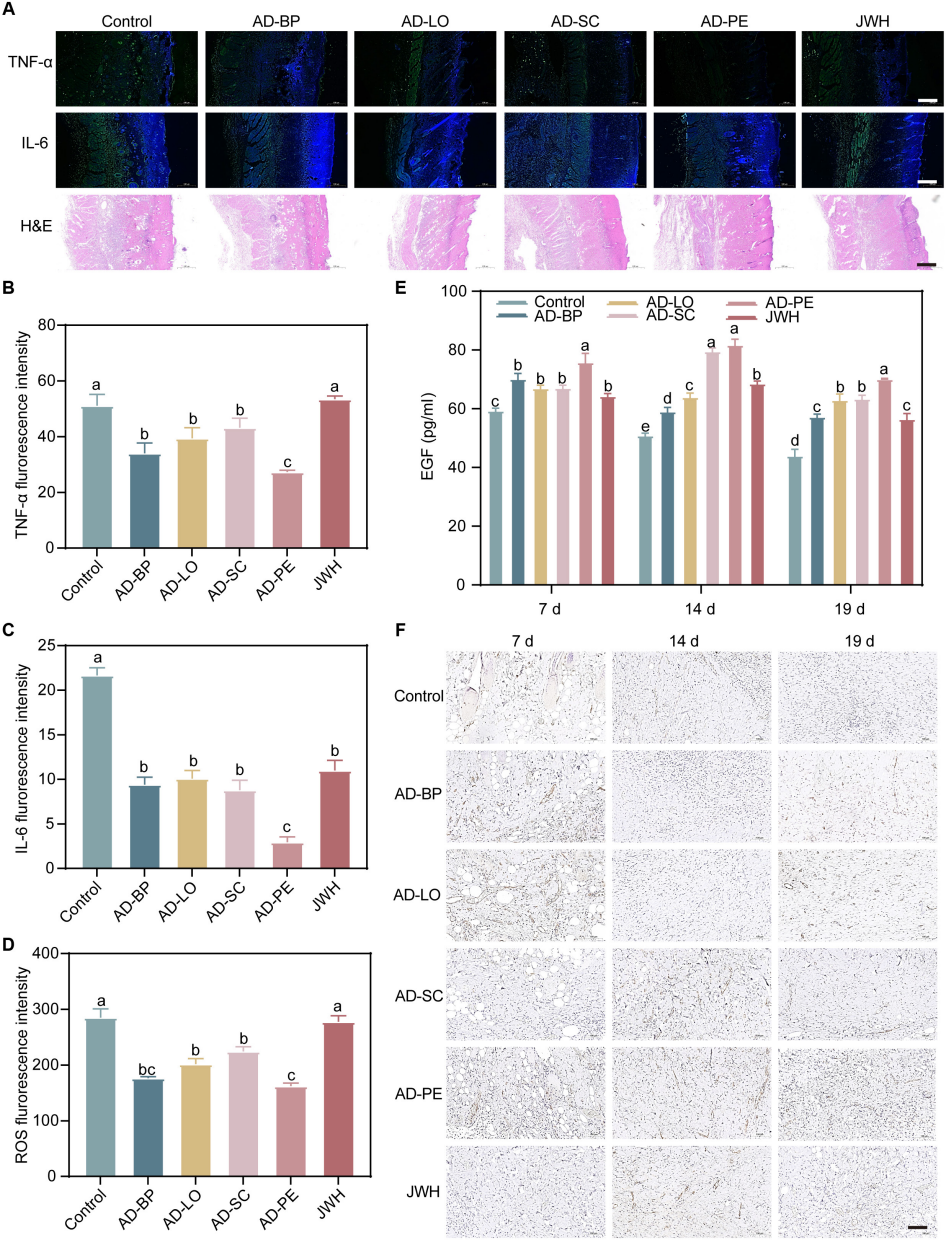
(A) Immunofluorescence staining images of TNF-α and IL-6, along with H&E staining. Scale bar, 500 μm. Quantification of the relative fluorescence intensity of (B) TNF-α, (C) IL-6, and (D) ROS in skin wound tissues from different treatment groups on day 5. (E) EGF of skin wound tissues in response to different groups on days 7, 14, and 19. Different letters indicate the significant difference (*P* < 0.05). Immunohistochemistry staining of (F) CD31. Scale bar, 100 μm.

Deep second-degree burn wounds are characterized by the infiltration of large numbers of inflammatory cells, particularly neutrophils, which leads to elevated levels of pro-inflammatory cytokines and excessive ROS production. This intense inflammatory response can disrupt the normal healing process by hindering the transition from the inflammatory phase to the proliferative phase. Elevated expression of pro-inflammatory cytokines such as TNF-α and IL-6 during wound healing may result in persistent inflammation, impairing tissue regeneration and delaying healing progression [47]. After 5 d of treatment, the fluorescence intensity of TNF-α (Fig. 9A and B) and IL-6 (Fig. 9A and C) in the AD-PE group was significantly lower than in the other groups. This indicated that AD-PE could synergistically enhance the efficacy of its components, effectively reducing the inflammatory response and promoting wound healing.

ROS is a key factor in the development of inflammation and chronic infections, with excessive accumulation leading to lipid peroxidation, cellular damage, and delayed wound healing [48]. Studies have shown that scavenging local ROS can effectively suppress inflammation, reduce tissue damage, and accelerate the healing process [49]. On day 5 post-injury, the ROS levels in the wound tissues of each group were assessed. After treatment with AD-PE, the ROS levels in the burn wound significantly decreased, indicating that the incorporation of AD-BP greatly enhanced ROS scavenging (Fig. 9D).

The role of EGF in skin wound healing has been extensively studied. It can stimulate the proliferation of epithelial cells, endothelial cells, and fibroblasts, thereby accelerating the healing process of burn wounds [50]. ELISA was used to measure the EGF levels in the wound tissue. On days 14 and 19, the EGF content in the AD-PE treatment group was significantly higher than in the other groups (Fig. 9E). This indicated that AD-PE could promote epidermal regeneration and accelerate the healing process of burn wounds by enhancing EGF secretion. Neovascularization is also a crucial factor in burn wound healing, as it facilitates dermal regeneration and the formation of intact skin [43]. Vascularization was assessed using the endothelial-specific marker CD31. On day 14, the AD-PE treatment group exhibited elevated expression of CD31 in the wound area, while the other groups showed only minimal expression (Fig. 9F). This indicated that the AD-PE group formed new blood vessels capable of supplying nutrients and oxygen necessary for cell proliferation [26]. On day 19 post-injury, a decrease in vascular density was observed along with increased collagen deposition and extended healing time, indicating that the wound had nearly completed the healing process (Fig. 9F). This suggested that AD-PE could maximally promote angiogenesis, thereby facilitating wound repair.

## Conclusion

In summary, AD-SC microgel solid particles were used to stabilize AD-LO, resulting in the preparation of a Pickering emulsion loaded with antioxidant peptides. The AD-PE formulation exhibited excellent stability, as confirmed by various analyses including storage tests, centrifugation, ζ-potential, and particle size measurements. Rheological evaluations demonstrated that the emulsion possesses appropriate viscosity for wound applications, while the antioxidant peptides enabled controlled release. In vitro assays revealed that AD-PE and its components possess excellent ROS scavenging and anti-inflammatory properties, with superior biocompatibility. The synergistic effects of the emulsion components resulted in enhanced wound healing, as demonstrated by complete healing and tissue regeneration, including the growth of epidermal tissue, blood vessels, and hair follicles, by day 19. These findings suggested that AD-PE effectively mitigated oxidative stress and inflammation, promoting rapid wound closure and tissue repair. These results highlighted the potential of AD-PE as a multifunctional wound dressing, offering a promising solution for addressing complex wound healing challenges.

## Ethical Approval

The animal experiments involving Sprague–Dawley rats and Chinese giant salamanders were conducted in compliance with the ethical guidelines for animal research established by the institution. All procedures adhered strictly to both institutional and national regulations concerning the care and use of laboratory animals, and all efforts were made to minimize animal suffering throughout the study. The animal maintenance and handling protocols were approved by the Guiyang University Experimental Animal Ethics Committee (approval number: GYU-BEE-2022-02).

## Data Availability

The data that support the findings of this study are available from the corresponding author upon reasonable request. All relevant data generated or analyzed during this study are included in this published article.
