# Supplementary material for: Biocompatible Pickering Emulsions from Andrias davidianus Byproducts for Promoting Burn Wound Healing
Source: Biomater Res. 2025 Aug 19;29:0233. doi: 10.34133/bmr.0233 (PMC12364539; doi:10.34133/bmr.0233)
Supplement: Supplementary 1 — Table S1 [file bmr.0233.f1.docx]

**Supplementary Table S1. List of Reviewed Articles**

| **No.** | **Title** | **Journal** | **Year** | **Relevance** |
| --- | --- | --- | --- | --- |
| 1 | Immunohistochemical staining of skin‑expressed proteins to identify exfoliated epidermal cells for forensic purposes | *Forensic Sci Int* | 2019 | Demonstrates advanced histological methods for skin analysis, informing our tissue characterization. |
| 2 | Bromelain‑loaded chitosan nanofibers prepared by electrospinning method for burn wound healing in animal models | *Life Sci* | 2019 | Provides a comparative approach to burn dressings incorporating proteolytic enzymes. |
| 3 | Collagens made from giant salamander (Andrias davidianus) skin and their odorants | *Food Chem* | 2021 | Details extraction and properties of A. davidianus collagen, directly relevant to our biomaterial source. |
| 4 | Stabilized collagen matrix dressing improves wound macrophage function and epithelialization | *FASEB J* | 2019 | Highlights the role of collagen dressings in modulating inflammation and cell migration. |
| 5 | Construction of a dual‑component hydrogel matrix for 3D biomimetic skin based on photo‑crosslinked chondroitin sulfate/collagen | *Int. J. Biol. Macromol.* | 2024 | Illustrates composite hydrogel strategies for skin regeneration under oxidative stress. |
| 6 | What is the role of peptide fragments of collagen I and IV in health and disease? | *Life Sci* | 2019 | Reviews bioactivity of collagen peptides, supporting our choice of antioxidant peptide components. |
| 7 | Chitosan: Whey Protein Isolate: An Effective Emulsifier for Stabilization of Squalene Based Emulsions | *Waste Biomass Valorization* | 2019 | Informs our design of Pickering emulsions for wound‑healing applications. |
| 8 | Antimicrobial hydrogel with multiple pH‑responsiveness for infected burn wound healing | *Nano Research* | 2023 | Offers insights into multifunctional hydrogels combining antimicrobial and antioxidant functions. |
| 9 | Peptide/protein hydrolysate and their derivatives: Their role as emulsifying agents for enhancement of physical and oxidative stability of emulsions | *Trends Food Sci. Technol.* | 2022 | Underpins the selection of antioxidant peptides for stabilizing Pickering emulsions. |
| 10 | Fabrication and characterization of Chinese giant salamander skin composite collagen sponge as a high‑strength rapid hemostatic material | *J. Biomater. Sci. Polym. Ed.* | 2018 | Demonstrates a hemostatic application of A. davidianus skin collagen, complementary to our hemostatic goal. |
| 11 | Collagen in Wound Healing | *Bioengineering (Basel)* | 2021 | Comprehensive review of collagen’s roles and mechanisms in wound repair, underpinning our choice of collagen-based materials. |
| 12 | Growth Factors, Reactive Oxygen Species, and Metformin—Promoters of the Wound Healing Process in Burns? | *International Journal of Molecular Sciences* | 2021 | Discusses the interplay between growth factors, ROS, and metformin in burn healing, supporting our focus on oxidative stress modulation. |
| 13 | Development and Use of Biomaterials as Wound Healing Therapies | *Burns & Trauma* | 2019 | Reviews current biomaterial strategies for wound management, providing context for our Pickering emulsion design. |
| 14 | Bioactive Bacterial Cellulose Wound Dressings for Burns with Collagen In‑Situ and Chitosan Ex‑Situ Impregnation | *International Journal of Biological Macromolecules* | 2023 | Describes composite dressings combining collagen and chitosan for burn wounds, informing our material formulation. |
| 15 | Innate Immune System Response to Burn Damage—Focus on Cytokine Alteration | *International Journal of Molecular Sciences* | 2022 | Analyzes cytokine changes after burn injury, informing our understanding of inflammation dynamics in wound healing. |
| 16 | Biological Activity of Propolis Ointment with the Addition of 1% Nanosilver in the Treatment of Experimentally‑Evoked Burn Wounds | *Polymers (Basel)* | 2021 | Evaluates antimicrobial and healing effects of propolis‑nanosilver dressings, relevant to our multifunctional material approach. |
| 17 | Advanced Multilayer Composite Dressing with Co‑Delivery of Gelsevirine and Silk Fibroin for Burn Wound Healing | *Composites Part B: Engineering* | 2023 | Demonstrates a layered dressing system combining bioactive compounds, supporting our emulsion‑based release strategy. |
| 18 | Enzyme‑Polymer Conjugates as Robust Pickering Interfacial Biocatalysts for Efficient Biotransformations and One‑Pot Cascade Reactions | *Angewandte Chemie International Edition* | 2018 | Describes Pickering stabilization and biocatalysis, guiding our emulsion design and stabilization rationale. |
| 19 | Novel Alginate/Hydroxyethyl Cellulose/Hydroxyapatite Composite Scaffold for Bone Regeneration: In Vitro Cell Viability and Proliferation of Human Mesenchymal Stem Cells | *International Journal of Biological Macromolecules* | 2018 | Presents composite scaffold fabrication and cell compatibility assays, relevant to our materials’ biocompatibility testing. |
| 20 | Revealing the Aggregation Behaviors of Mesostructured Collagen by the Evaluation of Reconstituted Collagen Performance | *Food Hydrocolloids* | 2022 | Explores collagen aggregation and performance, underpinning our choice of A. davidianus collagen microgels. |
| 21 | In Situ Formed Anti‑Inflammatory Hydrogel Loading Plasmid DNA Encoding VEGF for Burn Wound Healing | *Acta Biomaterialia* | 2019 | Reports on in situ hydrogel formation and gene‑delivery for angiogenesis, complementing our growth factor modulation approach. |
| 22 | Chitosan‑Based Multifunctional Hydrogel for Sequential Wound Inflammation Elimination, Infection Inhibition, and Wound Healing | *International Journal of Biological Macromolecules* | 2023 | Details a chitosan hydrogel with staged therapeutic functions, aligning with our focus on sequential ROS scavenging and anti‑inflammation. |
| 23 | Spindle‑Like Zinc Silicate Nanoparticles Accelerating Innervated and Vascularized Skin Burn Wound Healing | *Advanced Healthcare Materials* | 2022 | Demonstrates nanoparticle‑mediated angiogenesis and neurogenesis, informing our evaluation of vascular responses in healing. |
| 24 | Polypeptides‑Drug Conjugates for Anticancer Therapy | *Advanced Healthcare Materials* | 2021 | Reviews design of polypeptide conjugates, relevant to our approach of peptide‑based antioxidant delivery. |
| 25 | A Specific Peptide with Calcium Chelating Capacity Isolated from Whey Protein Hydrolysate | *Journal of Functional Foods* | 2014 | Characterizes peptide chelation properties, supporting our selection of AD‑BP for enhancing bioactivity in Pickering emulsions. |
| 26 | A Review of Recent Progress on Collagen‑Based Biomaterials | *Advanced Healthcare Materials* | 2023 | Summarizes advances in collagen scaffolds, providing comprehensive context for our A. davidianus collagen application. |
| 27 | Healing Properties of Epidermal Growth Factor and Tocotrienol‑Rich Fraction in Deep Partial‑Thickness Experimental Burn Wounds | *Antioxidants* | 2020 | Demonstrates synergistic EGF and antioxidant effects in burn healing, supporting our focus on EGF secretion enhancement. |
| 28 | Therapeutic Potential of Propolis in Alleviating Inflammatory Response and Promoting Wound Healing in Skin Burn | *Phytotherapy Research* | 2023 | Reviews propolis anti‑inflammatory mechanisms, informing our comparison of natural antioxidant sources. |
| 29 | Hydrothermal Extraction and Thorough Characterization of Carrageenans and Proteins from Gigartina pistillata | *Food Hydrocolloids* | 2024 | Details extraction and functionality of marine hydrocolloids, offering parallels to our extraction of A. davidianus byproducts. |
| 30 | Anti‑Dehydration and Rapid Trigger‑Detachable Multifunctional Hydrogels Promote Scarless Therapeutics of Deep Burn | *Advanced Functional Materials* | 2023 | Presents multifunctional hydrogel design for scarless healing, guiding our material development priorities. |
| 31 | Platelet Extracellular Vesicles Loaded Gelatine Hydrogels for Wound Care | *Advanced Healthcare Materials* | 2025 | Describes vesicle‑loaded hydrogel for wound care, informing our hydrogel’s delivery and regenerative strategies. |
| 32 | Biodegradable and injectable poly(vinyl alcohol) microspheres in silk sericin‑based hydrogel for the controlled release of antimicrobials: application to deep full‑thickness burn wound healing | *Advanced Composites and Hybrid Materials* | 2022 | Demonstrates microsphere‑hydrogel composites for controlled antimicrobial delivery, paralleling our Pickering emulsion approach. |
| 33 | Injectable and biofunctionalized fibrin hydrogels co‑embedded with stem cells induce hair follicle genesis | *Regenerative Biomaterials* | 2023 | Explores fibrin‑stem cell hydrogels for tissue regeneration, relevant to our evaluation of regenerative potential. |
| 34 | A robust, one‑pot synthesis of highly mechanical and recoverable double network hydrogels using thermoreversible sol‑gel polysaccharide | *Advanced Materials* | 2013 | Provides methodology for durable double‑network hydrogels, guiding our materials design for mechanical stability. |
| 35 | Controlled Release of Ceria and Ferric Oxide Nanoparticles via Collagen Hydrogel for Enhanced Osteoarthritis Therapy | *Advanced Healthcare Materials* | 2024 | Details nanoparticle release from collagen hydrogel, relevant to our ROS‑scavenging peptide delivery strategy. |
| 36 | Dynamic Covalent Dextran Hydrogels as Injectable, Self‑Adjuvating Peptide Vaccine Depots | *ACS Chemical Biology* | 2023 | Describes dextran hydrogels for peptide delivery, informing our Pickering formulation and bioactivity retention. |
| 37 | “Monitor‑and‑treat” that integrates bacterio‑therapeutics and bio‑optics for infected wound management | *Bioactive Materials* | 2025 | Presents integrated therapeutic‑optical hydrogel, offering insights into multifunctional wound dressing design. |
| 38 | Superb Silk Hydrogels with High Adaptability, Bioactivity, and Versatility Enabled by Photo‑Cross‑Linking | *ACS Nano* | 2024 | Reports on photo‑crosslinked silk hydrogels, relevant to our hydrogel crosslinking and functionalization methods. |
| 39 | Human hair keratin and its‑based biomaterials for biomedical applications | *Tissue Engineering and Regenerative Medicine* | 2014 | Reviews keratin biomaterials, providing context for alternative protein‑based dressings. |
| 40 | Trojan Horse Bioheterojunction Empowers Adhesive Hydrogel with Robust Antibacterial Activity and Sensing Capacity for Infected Cutaneous Regeneration | *Nano Letters* | 2024 | Introduces multifunctional adhesive hydrogel with sensing, aligning with our design goals for responsive materials. |
| 41 | All‑natural injectable hydrogel with self‑healing and antibacterial properties for wound dressing | *Cellulose* | 2020 | Details self‑healing, antibacterial hydrogel, supporting our scaffold’s design principles. |
| 42 | Multifunctional Hydrogel Based on Silk Fibroin Promotes Tissue Repair and Regeneration | *Advanced Functional Materials* | 2024 | Explores silk fibroin hydrogel for tissue regeneration, relevant to our evaluation of regenerative potential. |
| 43 | Self‑Cross‑Linked Oxidized Sodium Alginate/Gelatin/Halloysite Hydrogel as Injectable, Adhesive, Antibacterial Dressing for Hemostasis | *ACS Sustainable Chemistry & Engineering* | 2024 | Describes self‑cross‑linked hydrogel for hemostasis and antibacterial action, informing our material’s multifunctionality. |
| 44 | Protein‑based functional hydrogel improves cutaneous nerve repair and diabetic wound healing | *Nano Research* | 2025 | Reports on protein‑hydrogel for nerve repair and wound healing, relevant to our regenerative and cytocompatibility assessments. |
| 45 | Oral microsphere formulation of M2 macrophage‑mimetic Janus nanomotor for targeted therapy of ulcerative colitis | *Science Advances* | 2024 | Though focused on gastrointestinal therapy, this Janus nanomotor strategy informs our design of multifunctional delivery vehicles. |
| 46 | beta‑Glucan‑Based Wet Dressing for Cutaneous Wound Healing | *Advanced Wound Care (New Rochelle)* | 2019 | Details beta‑glucan dressings, relevant to polysaccharide‑based component selection in our system. |
| 47 | Cofunctionalization of Macroporous Dextran Hydrogels with Adhesive Peptides and Growth Factors Enables Vascular Spheroid Sprouting | *ACS Biomaterials Science & Engineering* | 2024 | Demonstrates peptide/growth factor functionalization, guiding our collaborative approach to EGF secretion enhancement. |
| 48 | Nanocellulose‑assisted 3D‑printable, transparent, bio‑adhesive, conductive, and biocompatible hydrogels as sensors and moist electric generators | *Carbohydrate Polymers* | 2023 | Explores nanocellulose hydrogels for sensing and healing, paralleling our interest in multifunctional material properties. |
| 49 | Silk fibroin‑based hydrogels for cartilage organoids in osteoarthritis treatment | *Theranostics* | 2025 | Reports on silk fibroin hydrogels for cartilage regeneration, supporting our broader hydrogel application context. |
| 50 | Cellulose‑Based Dual‑Network Conductive Hydrogel with Exceptional Adhesion | *Advanced Functional Materials* | 2024 | Describes conductive, adhesive cellulose hydrogel, informing our selection of bioactive polysaccharide components. |
| 51 | Injectable hyaluronate/collagen hydrogel with enhanced safety and efficacy for facial rejuvenation | *Collagen and Leather* | 2024 | Demonstrates injectable collagen–hyaluronate hydrogel safety and performance, informing our biocompatibility considerations for A. davidianus collagen systems. |
| 52 | Antibacterial polysaccharide-based hydrogel dressing containing plant essential oil for burn wound healing | *Burns & Trauma* | 2021 | Reports on essential-oil–loaded polysaccharide hydrogel antimicrobial efficacy, guiding our multifunctional emulsion design. |
| 53 | A drug‑free cardiovascular stent functionalized with tailored collagen supports in‑situ healing of vascular tissues | *Nature Communications* | 2024 | Illustrates collagen functionalization for tissue repair, supporting our use of A. davidianus collagen for regenerative applications. |
| 54 | A General and Convenient Peptide Self‑Assembling Mechanism for Developing Supramolecular Versatile Nanomaterials Based on The Biosynthetic Hybrid Amyloid‑Resilin Protein | *Advanced Materials* | 2024 | Describes peptide self‑assembly strategies, informing our approach to isolate and deploy AD‑BP peptides in Pickering emulsions. |
| 55 | Effect of sodium alginate-based hydrogel loaded with lutein on gut microbiota and inflammatory response in DSS-induced colitis mice | *Food Science and Human Wellness* | 2023 | Though focused on colitis, elucidates alginate hydrogel–lutein interactions and anti‑inflammatory effects relevant to oxidative stress modulation. |
| 56 | A natural polymer‑based hydrogel with shape controllability and high toughness and its application to efficient osteochondral regeneration | *Materials Horizons* | 2023 | Presents polymer hydrogel mechanics and regenerative performance, relevant to our design of mechanically robust wound dressings. |
| 57 | Molecular self‑assembly strategy tuning a dry crosslinking protein patch for biocompatible and biodegradable haemostatic sealing | *Nature Communications* | 2025 | Demonstrates in situ self‑assembling collagen patches, paralleling our strategy for haemostatic AD‑PE materials. |
| 58 | Effects of secondary cross-linking on the physicochemical properties of sodium alginate–hydrogel and in vitro release of anthocyanins | *International Journal of Biological Macromolecules* | 2024 | Examines crosslinking impact on alginate hydrogel release profiles, guiding our optimization of Pickering emulsion stability and release kinetics. |
| 59 | Characterization of a novel konjac glucomannan film incorporated with Pickering emulsions: Effect of the emulsion particle sizes | *International Journal of Biological Macromolecules* | 2021 | Reports Pickering emulsion size effects in polysaccharide films, directly paralleling our emulsion stabilization studies. |
| 60 | ζ Potential as a Measure of Asphalt Emulsion Stability | *Energy & Fuels* | 2020 | Provides foundational methods for zeta‑potential measurement in emulsions, applicable to our characterization of AD‑PE emulsions. |
| 61 | Colloid properties of hydrophobic modified alginate: Surface tension, ζ-potential, viscosity and emulsification | *Carbohydrate Polymers* | 2018 | Details physicochemical parameter measurements for alginate emulsions, informing our emulsion property analyses. |
| 62 | Relationship between the adsorption behavior at the polyol‑oil interface and the emulsion features | *Colloids and Surfaces A: Physicochemical and Engineering Aspects* | 2024 | Explores interfacial adsorption phenomena, underpinning our understanding of solid‑stabilized Pickering emulsions. |
| 63 | Fabrication and characterization of acid soluble collagen stabilized Pickering emulsions | *Food Hydrocolloids* | 2020 | Demonstrates collagen‑based Pickering emulsion formation, directly analogous to our AD‑SC microgel–stabilized systems. |
| 64 | Collagen and gelatin: Structure, properties, and applications in food industry | *International Journal of Biological Macromolecules* | 2024 | Reviews food‑grade collagen properties, providing context for our biomedical-grade A. davidianus collagen characterization. |
| 65 | A Modified Collagen Dressing Induces Transition of Inflammatory to Reparative Phenotype of Wound Macrophages | *Scientific Reports* | 2019 | Demonstrates collagen dressing immunomodulatory effects, supporting our focus on antioxidant peptides to modulate inflammation. |
| 66 | A Bioinspired Medical Adhesive Derived from Skin Secretion of Andrias davidianus for Wound Healing | *Advanced Functional Materials* | 2019 | Reports on A. davidianus skin‑derived adhesive, directly relevant to our exploration of salamander byproducts for wound care. |
| 67 | Soft microgels as Pickering emulsion stabilisers: role of particle deformability | *Soft Matter* | 2011 | Discusses microgel deformability in Pickering stabilization, guiding our use of AD‑SC microgels for enhanced emulsion stability. |
| 68 | Antioxidant activities of peptide fractions derived from freshwater mussel protein using ultrasound‑assisted enzymatic hydrolysis | *Czech Journal of Food Sciences* | 2017 | Details marine peptide antioxidant assays, informing our measurement of AD‑BP ROS‑scavenging capacity. |
| 69 | Sustainable Approach of Functional Biomaterials–Tissue Engineering for Skin Burn Treatment: A Comprehensive Review | *Pharmaceuticals* | 2023 | Provides broad overview of biomaterial strategies for burn treatment, contextualizing our multifunctional emulsion approach. |
| 70 | Recent developments in Pickering emulsions for biomedical applications | *Current Opinion in Colloid & Interface Science* | 2019 | Reviews biomedical Pickering emulsion advances, validating our choice of Pickering strategy for localized therapeutic delivery. |
| 71 | Pickering emulsions: Versatility of colloidal particles and recent applications | *Current Opinion in Colloid & Interface Science* | 2020 | Reviews colloidal particle–stabilized emulsions, informing our choice and design of Pickering systems for biomedical use. |
| 72 | Molecular Mechanisms Related to Burns, Burn Wound Healing and Scarring | *International Journal of Molecular Sciences* | 2023 | Summarizes burn pathophysiology and scarring mechanisms, providing background for our therapeutic strategy. |
| 73 | An Adhesive Bioink toward Biofabrication under Wet Conditions | *Small* | 2023 | Describes adhesive bioinks for wet environments, paralleling challenges in formulating wound dressings for moist wounds. |
| 74 | A barrier against reactive oxygen species: chitosan/acellular dermal matrix scaffold enhances stem cell retention and improves cutaneous wound healing | *Stem Cell Research & Therapy* | 2020 | Demonstrates ROS‑protective scaffold supporting cell retention, aligning with our focus on oxidative stress mitigation. |
| 75 | The Collagen Suprafamily: From Biosynthesis to Advanced Biomaterial Development | *Advanced Materials* | 2018 | Provides comprehensive review of collagen types and biomaterial applications, contextualizing our use of A. davidianus collagen. |
| 76 | Radix Salvia miltiorrhiza Ameliorates Burn Injuries by Reducing Inflammation and Promoting Wound Healing | *Journal of Inflammation Research* | 2023 | Shows herbal extract’s anti‑inflammatory and healing effects, supporting our natural‐product approach to inflammation control. |
| 77 | Antioxidant peptides from edible aquatic animals: Preparation method, mechanism of action, and structure‑activity relationships | *Food Chemistry* | 2023 | Reviews aquatic‐derived peptides, underpinning selection and characterization of AD‑BP antioxidant fractions. |
| 78 | Anti‑inflammation biomaterial platforms for chronic wound healing | *Biomaterials Science* | 2021 | Surveys biomaterials designed to modulate inflammation, guiding our integration of antioxidant and anti‑inflammatory functions. |
| 79 | Identification and Active Evaluation of Antioxidant Peptides from Protein Hydrolysates of Skipjack Tuna (Katsuwonus pelamis) Head | *Antioxidants* | 2019 | Illustrates identification and testing of marine antioxidant peptides, analogous to our AD‑BP evaluation. |
| 80 | A Bioinspired Hemostatic Powder Derived from the Skin Secretion of Andrias davidianus for Rapid Hemostasis and Intraoral Wound Healing | *Small* | 2021 | Demonstrates salamander skin‐derived hemostatic powder, directly relevant to our exploration of salamander byproducts for wound care. |
| 81 | Correlation between Fatty Acid Profile and Anti‑Inflammatory Activity in Common Australian Seafood by‑Products | *Marine Drugs* | 2019 | Links marine oil composition to anti‑inflammatory efficacy, informing our use of AD‑LO for oxidative stress and inflammation control. |
| 82 | Fabrication and characterization of rice bran oil‐in‐water Pickering emulsion stabilized by cellulose nanocrystals | *Colloids and Surfaces A: Physicochemical and Engineering Aspects* | 2017 | Demonstrates plant‐oil Pickering emulsions, paralleling our formulation of AD‑LO stabilized by microgel particles. |
| 83 | Stability of protein particle based Pickering emulsions in various environments: Review on strategies to inhibit coalescence and oxidation | *Food Chemistry: X* | 2023 | Reviews stabilization strategies against coalescence and oxidation, guiding our selection of AD‑SC microgels for emulsion stability. |
| 84 | Adhesive Nanoparticle‑in‑Microgel System with ROS Scavenging Capability and Hemostatic Activity for Postoperative Adhesion Prevention | *Small* | 2024 | Presents ROS‑scavenging, hemostatic microgel systems, analogous to our AD‑PE multifunctional emulsion. |
| 85 | Performance of Quillaja bark saponin and β‑lactoglobulin mixtures on emulsion formation and stability | *Food Hydrocolloids* | 2017 | Evaluates saponin–protein emulsifiers, relevant to understanding biomacromolecule–stabilized Pickering emulsions. |
| 86 | Role of gel structure in controlling in vitro intestinal lipid digestion in whey protein emulsion gels | *Food Hydrocolloids* | 2017 | Explores gel microstructure effects on release/digestion, informing our analysis of peptide release from Pickering emulsions. |
| 87 | One‑Step Synthesis of Multifunctional Chitosan Hydrogel for Full‑Thickness Wound Closure and Healing | *Advanced Healthcare Materials* | 2022 | Describes chitosan hydrogel with integrated functionalities, paralleling our hydrogel component design. |
| 88 | A double‑network polysaccharide‑based composite hydrogel for skin wound healing | *Carbohydrate Polymers* | 2021 | Demonstrates dual‐network polysaccharide hydrogel efficacy in wound repair, guiding our composite material design. |
| 89 | Clinical Impact Upon Wound Healing and Inflammation in Moist, Wet, and Dry Environments | *Advanced Wound Care (New Rochelle)* | 2013 | Reviews impact of wound environment on healing, contextualizing our in vivo and in vitro experiments. |
| 90 | Paramylon hydrogel: A bioactive polysaccharides hydrogel that scavenges ROS and promotes angiogenesis for wound repair | *Carbohydrate Polymers* | 2022 | Reports polysaccharide hydrogel ROS scavenging and angiogenesis promotion, analogous to our AD‑PE antioxidant and regenerative functions. |
| 91 | Rod‑shaped polypeptide nanoparticles for siRNA delivery | *International Journal of Biological Macromolecules* | 2021 | Demonstrates polypeptide nanoparticle design for nucleic acid delivery, informing our understanding of peptide-based carrier systems. |
| 92 | Effects of (+)-catechin on a rice bran protein oil‑in‑water emulsion: Droplet size, zeta‑potential, emulsifying properties, and rheological behavior | *Food Hydrocolloids* | 2020 | Explores polyphenol–emulsion interactions and stability, guiding our assessment of antioxidant–emulsion compatibility. |
| 93 | Injectable, self‑healing, antibacterial, and hemostatic N,O‑carboxymethyl chitosan/oxidized chondroitin sulfate composite hydrogel for wound dressing | *Materials Science & Engineering C* | 2021 | Describes multifunctional chitosan‑based hydrogel, paralleling our design of hydrogels with self‑healing and hemostatic properties. |
| 94 | Pickering emulsion gel stabilized by octenylsuccinate quinoa starch granule as lutein carrier: Role of the gel network | *Food Chemistry* | 2020 | Reports on starch‑based Pickering gels for bioactive delivery, analogous to our use of microgel networks in emulsions. |
| 95 | *Andrias davidianus* Mucus‑Based Bioadhesive with Enhanced Adhesion and Wound Healing Properties | *ACS Applied Materials & Interfaces* | 2023 | Demonstrates salamander mucus adhesive and healing efficacy, directly relevant to our utilization of A. davidianus byproducts. |
| 96 | Novel Diabetic Foot Wound Dressing Based on Multifunctional Hydrogels with Extensive Temperature‑Tolerant, Durable, Adhesive, and Intrinsic Antibacterial Properties | *ACS Applied Materials & Interfaces* | 2021 | Details multifunctional hydrogel dressing performance under challenging conditions, informing our material robustness criteria. |
| 97 | Structural characterization and in vitro immunogenicity evaluation of amphibian‑derived collagen type II from the cartilage of Chinese Giant Salamander (Andrias davidianus) | *Journal of Biomaterials Science, Polymer Edition* | 2020 | Provides immunogenicity and structural data on A. davidianus collagen, supporting its safe use in our biomaterials. |
| 98 | Preparation, characterization and antioxidant effect of polypeptide mineral‑chelate from Yanbian cattle bone | *LWT – Food Science and Technology* | 2023 | Illustrates mineral‑chelate peptide antioxidant activity, analogous to our evaluation of AD‑BP antioxidant capacity. |
| 99 | Design of gel structures in water and oil phases for improved delivery of bioactive food ingredients | *Critical Reviews in Food Science and Nutrition* | 2019 | Reviews gel network design in biphasic systems, guiding our formulation of oil‑in‑water Pickering emulsions. |
| 100 | Burn‑injured skin is marked by a prolonged local acute inflammatory response of innate immune cells and pro‑inflammatory cytokines | *Frontiers in Immunology* | 2022 | Details prolonged inflammation in burn wounds, providing context for our anti‑inflammatory and antioxidant strategies. |
| 101 | Preparation of nano‑hydroxyapatite/chitosan/tilapia skin peptides hydrogels and its burn wound treatment | *International Journal of Biological Macromolecules* | 2021 | Combines marine peptides and chitosan hydrogel for burn healing, paralleling our multi‑component hydrogel approach. |
| 102 | Ameliorative effect of orally administered different linoleic acid/α-linolenic acid ratios in a mouse model of DNFB-induced atopic dermatitis | *Journal of Functional Foods* | 2020 | Examines dietary fatty acid ratios on inflammation, informing our consideration of lipid components in wound modulation. |
| 103 | Biological activity evaluation and identification of different molecular weight peptides from wheat germ albumin | *LWT – Food Science and Technology* | 2023 | Characterizes wheat germ peptides, supporting our comparative analysis of peptide size and antioxidant activity. |
| 104 | Design and evaluation of new wound dressings based on collagen‑cellulose derivatives | *Materials & Design* | 2023 | Presents collagen‑cellulose composite dressings, informing our selection of matrix materials for structural and functional performance. |
| 105 | Polypeptide‑based drug delivery systems for programmed release | *Biomaterials* | 2021 | Reviews polypeptide carrier strategies, guiding our AD‑BP delivery design within Pickering emulsions. |
| 106 | Molecular mechanism of palmitic acid and its derivatives in tumor progression | *Frontiers in Oncology* | 2023 | Although oncology‑focused, provides insight into fatty acid bioactivity, relevant to AD‑LO’s anti‑inflammatory roles. |
| 107 | Influence of interfacial compositions on the microstructure, physiochemical stability, lipid digestion and β‑carotene bioaccessibility of Pickering emulsions | *Food Hydrocolloids* | 2020 | Explores interfacial composition effects on emulsion function, directly applicable to our AD‑SC microgel–stabilized emulsions. |
| 108 | Cellulose nanofiber from pomelo spongy tissue as a novel particle stabilizer for Pickering emulsion | *International Journal of Biological Macromolecules* | 2023 | Demonstrates fruit‑fiber‑based particle stabilization, analogous to our microgel particle approach. |
| 109 | A novel Pickering emulsion stabilized solely by hydrophobic agar microgels | *Carbohydrate Polymers* | 2022 | Reports agar microgel–stabilized emulsions, providing parallels to our collagen microgel systems. |
| 110 | A physicochemical double cross‑linked multifunctional hydrogel for dynamic burn wound healing: shape adaptability, injectable self‑healing property and enhanced adhesion | *Biomaterials* | 2021 | Details dynamic hydrogel properties for burn healing, aligning with our hydrogel’s multifunctionality. |
| 111 | Characterisation of acid‑soluble and pepsin‑solubilised collagen from frog (Rana nigromaculata) skin | *International Journal of Biological Macromolecules* | 2017 | Provides extraction and characterization methods for amphibian collagen, supporting our collagen sourcing techniques. |
| 112 | A hydrogel derived from skin secretion of Andrias davidianus to facilitate bone regeneration | *Composites Part B: Engineering* | 2024 | Reports salamander secretion–based hydrogel for bone repair, highlighting the versatility of A. davidianus byproducts. |
| 113 | Extraction of fish oil from fish heads using ultra‑high pressure pre‑treatment prior to enzymatic hydrolysis | *Innovative Food Science & Emerging Technologies* | 2021 | Describes optimized fish oil extraction, informing our AD‑LO preparation and quality considerations. |
| 114 | Application of Collagen‑Based Hydrogel in Skin Wound Healing | *Gels* | 2023 | Reviews collagen hydrogel applications in skin repair, contextualizing our use of A. davidianus collagen. |
| 115 | A hemostatic sponge derived from skin secretion of Andrias davidianus and nanocellulose | *Chemical Engineering Journal* | 2021 | Demonstrates salamander secretion sponge hemostatic efficacy, complementing our multifunctional dressing design. |
| 116 | Injectable thermo‑sensitive and wide‑crack self‑healing hydrogel loaded with antibacterial anti‑inflammatory dipotassium glycyrrhizate for full‑thickness skin wound repair | *Acta Biomaterialia* | 2022 | Describes thermo‑responsive, self‑healing hydrogel with antibacterial and anti‑inflammatory functions, paralleling our AD‑PE design for complex wound environments. |
